# Mobile Health Biometrics to Enhance Exercise and Physical Activity Adherence in Type 2 Diabetes (MOTIVATE-T2D): a decentralised feasibility randomised controlled trial delivered across the UK and Canada

**DOI:** 10.1136/bmjopen-2024-092260

**Published:** 2025-03-26

**Authors:** Katie Hesketh, Jonathan Low, Robert Andrews, Sandra Blitz, Benjamin Buckley, Kaja Falkenhain, Jennifer Job, Charlotte A Jones, Helen Jones, Mary E Jung, Jonathan Little, Ceu Mateus, Sarah L Percival, Richard Pulsford, Catherine L Russon, Joel Singer, Victoria S Sprung, Alison M McManus, Matthew Cocks

**Affiliations:** 1 School of Sport, Exercise and Rehabilitation Sciences, University of Birmingham, Birmingham, UK; 2 Research Institute for Sport and Exercise Science, Liverpool John Moores University, Liverpool, UK; 3 School of Health and Exercise Sciences, The University of British Columbia, Kelowna, British Columbia, Canada; 4 Exeter Medical School, University of Exeter, Exeter, UK; 5 Department of Diabetes, Taunton and Somerset NHS Foundation Trust, Taunton, UK; 6 Centre for Advancing Health Outcomes, Vancouver, British Columbia, Canada; 7 Liverpool Centre for Cardiovascular Science at University of Liverpool, Liverpool John Moores University and Liverpool Heart & Chest Hospital, Liverpool, UK; 8 Pennington Biomedical Research Center, Louisiana State University, Baton Rouge, Louisiana, USA; 9 The Mater Research Institute, The University of Queensland, Brisbane, Queensland, Australia; 10 Faculty of Medicine, The University of British Columbia, Kelowna, British Columbia, Canada; 11 Health Economics, Division of Health Research, Lancaster University, Lancaster, UK; 12 Public Health and Sports Science, University of Exeter, Exeter, UK

**Keywords:** Exercise, Diabetes Mellitus, Type 2, Telemedicine, Digital Technology, eHealth, Feasibility Studies

## Abstract

**ABSTRACT:**

**Objectives:**

Assess the feasibility of a mobile health (mHealth)-supported home-delivered physical activity (PA) intervention (MOTIVATE-T2D) in people with recently diagnosed type 2 diabetes (T2D).

**Design:**

Feasibility multicentre, parallel group, randomised controlled trial (RCT).

**Setting:**

Participants were recruited from England and Canada using a decentralised design.

**Participants:**

Adults (40–75 years) recently diagnosed with T2D (5–24 months).

**Interventions:**

Participants were randomised 1:1 to intervention (MOTIVATE-T2D) or active control groups. Participants codesigned 6month- home-delivered, personalised, progressive PA programmes supported by virtual behavioural counselling. MOTIVATE-T2D used biofeedback from wearable technologies to support the programme. The active control group received the same intervention without wearables.

**Outcomes:**

The primary outcomes were recruitment rate, retention and adherence to purposeful exercise. Clinical data on effectiveness were collected as exploratory outcomes at baseline, 6 and 12 months, with HbA1c and systolic blood pressure (BP) proposed as primary outcomes for a future full RCT.

**Results:**

n=135 eligible participants expressed an interest in the trial, resulting in 125 participants randomised (age 55±9 years, 48% female, 81% white), a recruitment rate of 93%. Retention at 12 months was 82%. MOTIVATE-T2D participants were more likely to start (OR 10.4, CI 3.4 to 32.1) and maintain purposeful exercise at 6 (OR 7.1, CI 3.2 to 15.7) and 12 months (OR 2.9, CI 1.2 to 7.4). Exploratory clinical outcomes showed a potential effect in favour of MOTIVATE-T2D, including proposed primary outcomes HbA1c and systolic BP (between-group mean differences: HbA1c: 6 months: −5% change from baseline, CI −10 to 2: 12 months: −2% change from baseline, CI −8 to −4; systolic BP: 6 months: −1 mm Hg, CI −5 to 3: 12 months: −4 mm Hg, CI −8 to 1).

**Conclusions:**

Our findings support the feasibility of delivering the MOTIVATE-T2D mHealth-supported PA intervention for people with recently diagnosed T2D and progression to a full RCT to examine its clinical and cost-effectiveness.

**Trial registration number:**

ISRCTN: 14335124; ClinicalTrials.gov: NCT0465353.

**STRENGTHS AND LIMITATIONS OF THIS STUDY**

- Our active control intervention was matched aside from the availability of technology in Mobile Health Biometrics to Enhance Exercise and Physical Activity Adherence in Type 2 Diabetes (MOTIVATE-T2D) to assess how the addition of mHealth technology can influence physical activity (PA).
- Acceptability and feasibility were established using a multidisciplinary approach drawing on quantitative, qualitative and trial procedure data.
- The effect of the interventions on PA was evaluated using two device-derived measurements. Providing objective data on purposeful exercise of moderate-to-vigorous intensities and daily lifestyle PA, which were both encouraged by the intervention and are known to be important determinants of health outcomes in T2D.
- This study was not designed or powered to definitively assess the efficacy or cost-effectiveness of the MOTIVATE-T2D intervention on clinical outcomes in people with newly diagnosed T2D.

## Introduction

 Increasing physical activity (PA), both through purposeful exercise and unstructured lifestyle behaviours is fundamental to the initial treatment of type 2 diabetes (T2D) and recommended in international guidelines.^1^,^3^ These guidelines draw on the known benefits of regular PA on glycaemia,^4^ the incidence of microvascular complications, cardiovascular events and all-cause mortality in people living with T2D.^5^ However, people with T2D tend to exhibit lower levels of PA compared with people who do not have diabetes.^6 7^ To address this gap, diabetes care pathways are increasingly prioritising the provision of personalised PA guidance for individuals recently diagnosed with T2D.^8 9^ However, given the poor adherence to existing PA interventions and strategies,^10^ innovative interventions which capitalise on the potential of new technologies are urgently needed to effectively support PA and health in people with T2D.

Recently, wearable technologies incorporating PA trackers have become popular to promote behaviour change in long-term conditions such as T2D.^11^ In particular, pedometers and accelerometers that provide biofeedback on ambulatory PA have been used as tools for self-monitoring or alongside more complex behavioural interventions.^12^ Use of PA trackers has been associated with increased PA in people with T2D,^13^ which can be maintained for up to 12 months.^14^ However, the effects of increasing PA via the use of PA trackers on outcomes relevant to the clinical management of T2D are unclear. An umbrella review found little evidence that PA trackers improve HbA1c in people with T2D.^15^ It has been hypothesised that these findings are due to PA trackers promoting low-intensity lifestyle PA (ie, accrual of PA through everyday activities) rather than more intense domains and/or purposeful exercise,^14^ crucial for improving glycaemic control.^4 16^

Accelerometers can support behaviour change by providing PA targets based on time spent in specific intensity categories (ie, light, moderate or vigorous). Intensity categories are delineated using threshold values derived from calibration studies, which examine the association between movement acceleration and energy expenditure.^17^ Generic targets based on such broad classifications of intensity are effective at encouraging general lifestyle PA, but the one-size-fits-all approach does not provide personalised formative feedback. Therefore, participants cannot use these targets to optimise intensity during purposeful exercise,^18^ which may be crucial for improving glycaemic control.^4^ Previous reports have also cited issues with using accelerometers to assess non-ambulatory activity (eg, cycling and resistance exercise), which may be promoted through purposeful exercise programmes.^19^ The latest generation smartwatches now incorporate heart rate (HR) monitors alongside accelerometers. HR monitoring has several advantages over accelerometers when targeting purposeful exercise of moderate-to-vigorous intensity. HR is the most accurate way to track the body’s response to PA, providing real-time objective personalised data that accounts for age, body mass and fitness level.^20^ HR also reflects intensity regardless of the type of activity performed.^21^ The Mobile Health Biometrics to Enhance Exercise and Physical Activity Adherence in Type 2 Diabetes (MOTIVATE-T2D) intervention was designed to combine accelerometery and HR monitoring as the most effective biofeedback tools to facilitate home-based PA, promoting both lifestyle PA and purposeful exercise of moderate-to-vigorous intensities known to influence clinical outcomes.^4^ Previous work suggests real-time HR biofeedback facilitates purposeful exercise by helping participants work at an intensity most likely to elicit health changes, fostering self-efficacy to engage through feelings of competence.^19^

Advances in mobile health (mHealth) technologies can also be used to target other barriers to home-delivered PA. One such barrier is that participants do not receive appropriate support from health providers in the time between scheduled meetings.^22^ MOTIVATE-T2D uses next-generation mHealth technology to share biometric data and facilitate remote communication between patients and health professionals. This aims to recreate the relationship between patients and health professionals experienced during supervised interventions, but with the advantage that communication is in the patient’s own environment at convenient times. Qualitative data suggests such remote feedback may encourage adherence to a PA programme, through enhanced relatedness between patients and health professionals.^19^ In summary, MOTIVATE-T2D combines the latest advances in biofeedback and data sharing to optimise a home-delivered behavioural counselling service. Together, the elements provide comprehensive support to participants, enabling them to codevelop personalised PA plans, which include lifestyle PA and purposeful exercise of moderate-to-vigorous intensities.

The primary aim of this study was to assess the feasibility of undertaking a subsequent definitive randomised controlled trial (RCT) to assess the clinical effectiveness and cost-effectiveness of the MOTIVATE-T2D intervention in people with recently diagnosed T2D. Specific objectives of the study were to:

1. Determine the proportion and characteristics of people with recently diagnosed T2D who would be willing to take part in an RCT (ie, recruitment rate).
2. Determine the number of participants retained at 12 months in both arms of the trial (ie, loss to follow-up).
3. Evaluate the acceptability of the intervention and determine the rates of adherence during and for 6 months after the completion of the intervention.
4. Estimate the precision of potential outcome measures required for sample size estimations for a future definitive RCT.
5. Pilot methods for collecting outcome measures, recruitment, randomisation, treatment, and follow-up.
6. Determine the availability and completeness of economic data.

## Methods

The trial is reported in accordance with the Consolidated Standards of Reporting Trials (CONSORT) extension for pilot trials.^23^ Our full trial protocol can be found elsewhere.^24^

### Study population and design

The MOTIVATE-T2D feasibility trial was a multicentre (UK and Canada), parallel group, RCT in adults recently diagnosed with T2D. Participants aged 40–75 years, diagnosed with T2D within the previous 5–24 months and managing their condition through lifestyle modification alone or metformin (stable dose for ≥3 months) were eligible. Exclusion criteria were HbA1c >86 mmol/mol, blood pressure >160/100 mm Hg, glucose-lowering agents other than metformin, unstable angina, myocardial infarction within the previous 3 months, transient ischaemic attack in the previous 6 months, heart failure New York Heart Association (NYHA) ≥class II, arrhythmia, healthcare professional advice against the increasing level of activity, pregnancy or planning to become pregnant, <6 months postpartum or stopped breastfeeding <1 month before recruitment, not owning a smartphone or not having a data plan or access to Wi-Fi and those who are currently meeting the recommended exercise guidelines for people with T2D (150 min of moderate intensity exercise per week).

Participants were randomised to active control or intervention (MOTIVATE-T2D) on a 1:1 basis following the completion of baseline assessments. Randomisation was stratified by the centre (UK or Canada), sex (male or female) and age (40–60 or 61–75 years) and was administered using a computer-generated random allocation sequence, created and administered by the Centre for Advancing Health Outcomes. Research staff responsible for the intervention and outcome measures and participants were aware of the allocation. However, the statistician undertaking the data analysis was blinded to treatment allocation (blinded analytic assessment). The trial conforms with the principles outlined in the Declaration of Helsinki and was approved in the UK by the South East Scotland Research Ethics Committee 01 (20/SS/0101) and in Canada by the Clinical Research Ethics Board of the University of British Columbia (H20-01936). All participants provided written informed consent.

Participants were recruited from (1) general practitioner (GP) database searches (UK only); (2) flyers provided to diabetes education sessions (Diabetes Education and Self Managment for Ongoing and Newly Diagnosed (DESMOND) and Expert Education versus Routine Treatment (X-PERT Health); both health professional-led structured diabetes education and self-management programmes; UK only); (3) adverts in clinical settings (LifeLabs and GP waiting rooms, Canada only); (4) local media adverts and classifieds (Canada only); (5) third-party online recruitment services (Lindus Health, UK; Trialfacts and HoneyBee Trials, Canada); (6) consent to contact database (Research For The Future, UK only) and (7) the study website.

### Interventions

Detailed descriptions of the active control and MOTIVATE-T2D interventions are published elsewhere.^24^ Briefly, by design, the active control and MOTIVATE-T2D interventions were matched aside from the availability of technology in MOTIVATE-T2D. Key aspects of both interventions and how they differed are presented in table 1.

**Table 1:** Intervention components

Intervention component	Shared features	Active control	MOTIVATE-T2D
Aims	Progressively increase purposeful exercise of moderate-to-vigorous intensities Increase daily lifestyle PA of any intensity		Supported by biofeedback and data sharing enabled by mHealth technologies: Smartwatch, featuring a 3D accelerometer and optical heart rate (HR) monitor Online coaching platform for the exercise specialist Web/smartphone app for participants
Behavioural counselling	Exercise specialist-led, one-to-one, virtual exercise consultations (Zoom) Five sessions: (1) prior to intervention, (2) approx. 1 week after first session, (3) 1 month, (4) 3 months, (5) 6 months		
Purposeful exercise programmes	Individualised action plans were codeveloped Individualised by allowing participants to choose the: Mode (eg, commuting, outdoor, indoor callisthenics or gym-based) Type (eg, walking, cycling, resistance exercise, interval-based exercise, classes, dance or sports) Initial duration Initial intensity Rate of progression	Action plan sent within a booklet containing: Training calendar/diary Progressive exercise guide Access to the trial website with exercise resources, including exercise videos	Action plan built within the coaching platform Training calendar Preset sessions prescribing the duration and intensity (measured through HR) of phases within each exercise session (ie, warm-up, workout and cool-down) Smartwatch providing real-time feedback on exercise intensity through HR zones During preset sessions, prescribed duration and intensity were displayed (via HR zones), with visual and haptic (vibration) alerts to coach participants Web/smartphone app: Access to the action plan Track exercise and PA achievements Participants rate enjoyment and provide written feedback following exercise sessions
Lifestyle PA	Advice on how to integrate physical activity into daily routines		Supported with a target on the smartwatch
Ongoing communication	Counselling sessions 3, 4 and 5 Review progress Update action plan Counselling session 5 Strategies for maintaining exercise and PA without support from the exercise specialist	Updated action plans sent after counselling sessions Participants received SMS text messages: Weekly during the first 3 months Biweekly during months 4–6 Prescripted, based on self-determination theory: modelled to target relatedness, competence and autonomy Participants could reply and engage with their exercise specialist Action plans could be updated based on discussions	Consultations allowed mHealth data to guide discussions Updated action plans within the coaching platform after counselling sessions Updated preset sessions Participants received SMS text messages: Following each recorded exercise session during month 1 Weekly during months 2–3 Biweekly months 4–6 Based on data gathered by the mHealth technologies (intensity and duration of sessions and participant enjoyment and feedback) Participants could reply and engage with their exercise specialist Action plans and preset sessions could be updated based on discussions

MOTIVATE-T2D, Mobile Health Biometrics to Enhance Exercise and Physical Activity Adherence in Type 2 Diabetes; PA, physical activity.

Participants in both groups codesigned their 6month PA- programmes with the aim of promoting two behaviours, namely (1) gradually increasing purposeful exercise of moderate-to-vigorous intensity, with a target of 150 min per week by the end of 6 months and (2) increasing daily lifestyle PA of any intensity. These aims were facilitated by an exercise specialist-led behavioural counselling service delivered virtually via phone or teleconferencing software, according to participant preference. The counselling supported participants to develop personalised PA programmes and provided regular feedback on their action plans to support them achieve their goals. MOTIVATE-T2D used biofeedback and data sharing enabled by mHealth technologies to support the development of personalised PA programmes and ongoing feedback. The mHealth technologies included a smartwatch, featuring a 3D accelerometer and optical HR monitor (Polar Ignite, Polar Electro), synced with an online coaching platform for the exercise specialist (Polar Flow for Coach, www.polar.com/coach) and web/smartphone app for participants (Polar Flow—Sync & Analyze).

### Ongoing management of diabetes

Throughout the trial, all participants remained under their diabetes specialist and continued with medication management according to the national guidelines (UK, National Institute for Health and Care Excellence guidance on management of diabetes (NG28), hypertension (NG136) and lipid modification (NG181); Canada, Diabetes Canada 2018 Clinical Practice Guidelines).^25^

### Outcome measurements

The following feasibility outcomes were collected: percentage of people eligible for the study; total recruitment rate and rate by recruitment strategies; attrition and loss to follow-up; completeness of outcome measures at baseline, 6 and 12 months.

*Acceptability of study participation and intervention* were assessed via virtual (Zoom) semistructured qualitative interviews. Interviews were planned with a purposeful sampling framework, considering gender, age and country. Immediately following baseline measures, eight participants (UK n=4, Canada n=4) and 20 participants at 6 months (UK n=11, Canada n=9) were interviewed regarding study participation. At 6 months, 25 participants (MOTIVATE-T2D n=14, UK n=8, Canada n=6; active control n=11, UK n=8, Canada n=3) were interviewed regarding intervention acceptability, with 21 (MOTIVATE-T2D n=12, UK n=8, Canada n=4; active control n=9, UK n=6, Canada n=3) of these participants interviewed again at 12 months.

*Adherence:* the intervention aimed to increase completion of purposeful exercise of moderate-to-vigorous intensities and unstructured lifestyle PA; as such, two methods for assessing these distinct factors were employed.

Optical HR monitoring (photoplethysmography) was used to record the dose of purposeful exercise throughout the 12-month trial. A blinded Polar Verity Sense (Polar Electro, Finland) was provided to active control participants for the duration of the trial. The MOTIVATE-T2D group used a Polar Verity Sense paired with the fitness watch, allowing HR to be visualised in real time. HR data were used to calculate (1) the frequency of exercise (number of sessions recorded); (2) adherence to prescribed exercise (per cent of 78 sessions achieved, based on prescribing three sessions per week for 26 weeks), (3) duration of exercise; (4) duration of moderate-to-vigorous intensity exercise (MVE, calculated by adding time in moderate, 50%–70% HR_max_, and time in vigorous, ≥70% HR_max_*2, intensity exercise); (5) training drop-off (defined as the week in which participants no longer completed any training sessions) and; (6) proportion of participants completing >150 min of MVE per week at least once during the last month of the intervention and follow-up period.

Lifestyle PA was measured in all participants using a GENEactive (Activinsights, Kimbolton, Cambridge, UK) triaxial accelerometer for 14 days at baseline, 6 and 12 months. Data were extracted using GENEActiv PC software (V.3.0_09.02.2015) and processed in R using the open-source package GGIR V.1.2–8 (https://cran.r-project.org/web/packages/GGIR/index.html)^26^ to explore accelerometer wear time and the proportion of participants who wore the device for at least 16 hours on: (1) 4 days including 1 weekend day; (2) 3 days including at least 1 weekend day; (3) 3 days irrespective of weekend days, and (4) 1 day. The time spent in activity intensities was established using published thresholds.^27^ The following metrics of PA were assessed: average weekly minutes of total PA (any intensity), and of light, moderate, vigorous and moderate-to-vigorous PA (MVPA) and MVPA recorded in ≥10 min bouts (MVPA10+).

*Fidelity:* exercise specialists logged all contact with participants, including (1) the number of counselling sessions attended; (2) the number of SMS text messages sent by participants to exercise specialists and (3) the number of exercise video views.

Clinical effectiveness outcomes proposed for a future trial were collected at baseline, 6 and 12 months as described previously ^24^(see online supplemental eFigure 1 for a schematic outlining the study timeline). The trial used a decentralised design where outcomes were measured using remote ‘home-based’ solutions. HbA1c and systolic blood pressure were collected as the proposed primary outcomes for a future RCT. A number of proposed secondary outcomes were collected, including height, weight, waist circumference, diastolic blood pressure, mean arterial pressure, blood lipids, generic health status (5-level EQ-5D),^28^ health-related quality of life (12-Item Short Form Health Survey (SF-12)),^29^ diabetes treatment satisfaction (Diabetes Treatment Satisfaction Questionnaire status version (DTSQs)),^30^ healthcare utilisation using a study-specific questionnaire (primary and secondary care contacts, social care contacts and relevant medication usage) and safety outcomes (serious adverse events). Change in diabetes treatment satisfaction (DTSQ change version)^31^ was assessed at 6 months only. Free-living glycaemia was assessed using a FreeStyle Libre Pro flash continuous glucose monitor (CGM, Abbott Diabetes Care, Alameda, CA, USA) worn for 14 days at baseline, 6 and 12 months. Core CGM endpoints outlined in a recent position statement^32^ were calculated using the web-based application Diametrics^33^ if participants provided ≥70% of data over 14 consecutive days. To explore if different ways of processing CGM data influenced data availability and outcomes, CGM endpoints were calculated based on alternative wear-time criteria, including ≥80% of data over 10 consecutive days and ≥80% of data over 7 consecutive days.^32^

*Protocol deviation:* the original protocol suggested HbA1c would be analysed by the Exeter Clinical Laboratory for the UK and Canadian samples. Due to logistical challenges with shipping, Canadian HbA1c was assessed by the University of British Columbia research team, using an Afinion 2 point-of-care analyzer (Alere Technologies, Oslo, Norway). UK and Canadian assessment of blood lipids was conducted as planned by the Exeter Clinical Laboratory.

### Statistical analysis

Our planned recruitment target of 120 participants (60 per arm) allowed us to achieve the feasibility aims and objectives of this study; that is, an estimate of attrition, estimates of the SD of the secondary outcomes to inform power calculations for a future definitive trial and enough participants for qualitative interviews. For more information, see our full trial protocol.^24^

We report the mean and SD for both groups for all outcomes at baseline, 6 and 12 months, and the model-derived estimated marginal means (with corresponding 95% CIs) for the within- and between-group effect estimates at 6 and 12 months. Effect estimates are based on intention to treat analyses and included all participants that had a baseline or a follow-up value. Data were analysed via constrained longitudinal data analysis using a linear mixed model with fixed effects for time points (baseline, 6 and 12 months), the interaction between time point and intervention group, and stratified allocation factors (sex, study site and age category) and a random effect for participants.

Given the feasibility nature of this trial, we do not report p values for the comparison of outcomes to baseline or between groups. CIs and minimal clinically important differences (MCIDs) are presented to suggest plausible evidence of effect for respective study arms.^34 35^ All analyses were conducted in R (V.4.3.1). Participant semi-structured interviews were conducted by BJRB, SCP and JJ, who were not otherwise involved in the intervention or study delivery. Interviews were audio recorded and transcribed verbatim using otter.ai software. Transcripts were analysed using a deductive coding and thematic analysis approach by experienced qualitative researchers (SCP, JJ and MEJ),^36^ using NVivo 12TM software and will be fully reported elsewhere.

### Patient and public involvement (PPI)

Patients were involved in the oversight of trial progress and conduct via representation at periodic Trial Delivery Group and Trial Steering Group meetings. Our patient representatives also provided opinions on the protocol and patient-facing documentation (eg, participant information sheet) during the set-up of the trial.

## Results

### Recruitment and retention of participants and acceptability of trial design

**Figure 1:**
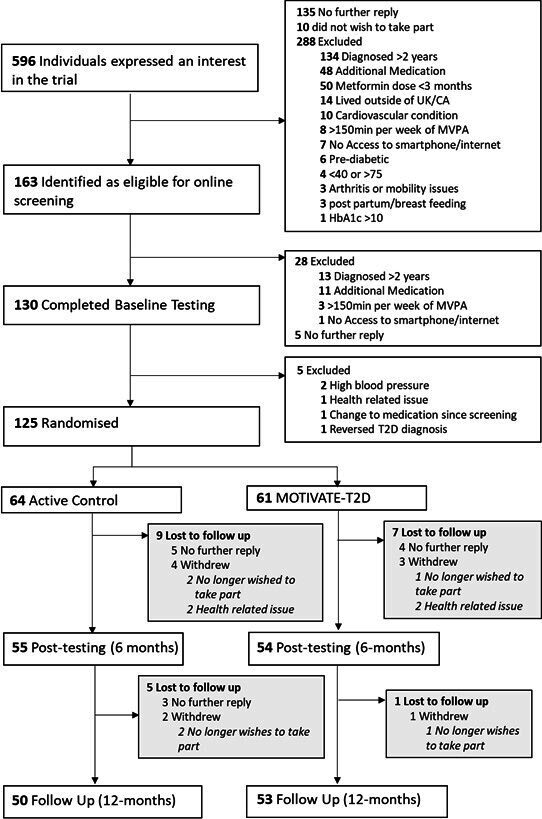
Consolidated Standards of Reporting Trials (CONSORT) flow chart. MVPA, moderate-to-vigorous physical activity; T2D, type 2 diabetes.

A CONSORT diagram showing participant flow through the study is shown in figure 1 (CONSORT diagrams separating UK and Canada are shown in online supplemental eFigures 2 and 3). Between January 2021 and December 2021, n=596 potential participants expressed an interest in the trial, of which n=321 (54% of those who initially enquired) were excluded, 140 (23%) had no further contact and 10 (2%) did not wish to take part, resulting in 125 participants (93% of eligible participants) randomised (MOTIVATE-T2D 61, active control 64; UK 62, Canada 63). The actual recruitment rate of 10.4 participants per month was in line with the forecast of 10 participants per month. Enrolment success appeared to be influenced by recruitment strategy (online supplemental eTable 1). For example, GP database searches were responsible for 94 expressions of interest and 44 participants randomised (47% of those who initially enquired), compared with third-party recruitment services, which were responsible for 327 expressions of interest and 38 participants randomised (12% of those who initially enquired). Demographics were also influenced by recruitment strategy (online supplemental eTable 2), with third-party online recruitment services recruiting more young people in full-time employment with education to higher level, but a more ethnically diverse population.

At 6- and 12-month follow-ups, 16 (13%) and 22 (18%) participants were lost to follow-up, respectively. There was evidence of imbalance in retention rate between study groups and country, with 8 (13%) MOTIVATE-T2D versus 14 (22%) active control and 9 (14%) UK versus 13 (21%) Canadian participants lost to follow-up. Participants had a high level of satisfaction with their participation in the trial (online supplemental eTable 3).

### Baseline characteristics

Baseline demographics and medication use are displayed in table 2 and online supplemental eTable 4, respectively (UK and Canada; online supplemental eTables 5–8). Baseline values for outcome measures are shown in tables3  4. Compared with active control, MOTIVATE-T2D included a higher proportion of participants with education to a further level and a lower proportion of participants who were single and living alone.

**Table 2:** Baseline demographic characteristics

	Total	Active control	MOTIVATE-T2D
N	125	64	61
Age, years, mean (SD)	55 (9)	54 (9)	55 (9)
Female, N (%)	60 (48)	31 (48)	29 (48)
Male, N (%)	65 (52)	33 (52)	32 (52)
Duration of T2D, months, mean (SD)	13 (6)	13 (6)	13 (6)
Ethnicity, N (%)			
White	101 (81)	52 (81)	49 (80)
African or Caribbean	5 (4)	2 (3)	3 (5)
Asian	14 (11)	8 (13)	6 (10)
Other or mixed	5 (4)	2 (3)	3 (5)
Marital status, living arrangements, N (%)			
Married, living with spouse	90 (72)	44 (69)	46 (75)
Married, living alone	1 (1)	0 (0)	1 (2)
Married, living arrangement unknown	1 (1)	0 (0)	1 (2)
Single, living alone	15 (12)	10 (16)	5 (8)
Single, living with others	3 (2)	2 (3)	1 (2)
Single, living arrangement unknown	1 (1)	1 (2)	0 (0)
Separated, living alone	9 (7)	5 (8)	4 (7)
Separated, living with spouse/partner	1 (1)	0 (0)	1 (2)
Widowed, living with spouse/partner	1 (1)	0 (0)	1 (2)
Widowed, living alone	2 (2)	1 (2)	1 (2)
Rather not say, living with spouse/partner	1 (1)	1 (2)	0 (0)
Educational attainment, N (%)			
Secondary	20 (16)	15 (23)	5 (8)
Further	49 (39)	20 (31)	29 (48)
Higher	56 (45)	29 (45)	27 (44)
Employment situation, N (%)			
Full-time	75 (60)	40 (63)	35 (57)
Part-time	11 (9)	4 (6)	7 (11)
Retired	21 (17)	11 (17)	10 (16)
Student	2 (2)	1 (2)	1 (2)
Voluntary/unpaid work	2 (2)	1 (2)	1 (2)
Stay-at-home mother/father	1 (1)	1 (2)	0 (0)
Unable: caring responsibility	1 (1)	0 (0)	1 (2)
Unable: ill health/disability	9 (7)	5 (8)	4 (7)
Unemployed	3 (2)	1 (2)	2 (3)

MOTIVATE-T2D, Mobile Health Biometrics to Enhance Exercise and Physical Activity Adherence in Type 2 Diabetes.

**Table 3:** Baseline and within- and between-group differences at 6- and 12-months follow-ups; HbA1c, anthropometrics, blood pressure, blood lipids, device-derived physical activity and continuous glucose monitoring variables

Outcome, unit (MCID)		Baseline, mean (SD)	Within-group difference(95% CI)*		Between-group difference(95% CI) *	
			6 months	12 months	6 months	12 months
HbA1c, mmol/mol (6%)	Active control	51 (11)	2 (–3 to 6)	2 (–3 to 7)	−5 (–10 to 2)	−2 (–8 to 4)
	MOTIVATE-T2D	50 (9)	−3 (7 to 1)	−1 (–5 to 4)		
			% change †			
Weight, kg (5 kg)	Active control	99.1 (23.6)	−2.9 (–4.4 to –1.4)	−2.7 (–4.2 to –1.2)	1.5 (–0.5 to 3.3)	1.2 (–0.9 to 3.3)
	MOTIVATE-T2D	98.0 (23.6)	−1.4 (–2.8 to 0.0)	−1.5 (–3.0 to –0.1)		
BMI, kg/m^2^	Active control	34.9 (8.4	−1.1 (–1.6 to –0.6)	−1.0 (–1.5 to –0.5)	0.6 (–0.1 to 1.2)	0.4 (–0.3 to 1.1)
	MOTIVATE-T2D	33.3 (6.1)	−0.5 (–1.0 to 0.0)	−0.6 (–1.1 to –0.1)		
Systolic BP, mm Hg (5 mm Hg)	Active control	130 (15)	−1 (–5 to 2)	0 (–3 to 4)	−1 (–5 to 3)	−4 (–8 to 1)
	MOTIVATE-T2D	127 (14)	−3 (–6 to 1)	−4 (–7 to 0)		
Diastolic BP, mm Hg (2 mm Hg)	Active control	82 (9)	−1 (–3 to 1)	−2 (–4 to 0)	0 (–2 to 3)	0 (–3 to 3)
	MOTIVATE-T2D	81 (9)	−1 (–3 to 1)	−2 (–4 to 0)		
MAP, mm Hg (2 mm Hg)	Active control	98 (10)	−1 (–3 to 1)	−1 (–3 to 1)	0 (–3 to 3)	−1 (–4 to 2)
	MOTIVATE-T2D	97 (10)	−1 (–3 to 1)	−2 (–4 to 0)		
Total Chol, mmol/L (0.3 mmol/L)	Active control	5.3 (1.5)	−0.2 (–0.5 to 0.1)	0.0 (–0.3 to 0.4)	0.1 (–0.3 to 0.5)	−0.3 (–0.7 to 0.1)
	MOTIVATE-T2D	5.4 (1.3)	−0.1 (–0.5 to 0.2)	−0.3 (–0.6 to 0.0)		
HDL Chol, mmol/L (0.1 mmol/L)	Active control	1.2 (0.3)	0.0 (0.0 to 0.1)	0.0 (0.0 to 0.1)	0.0 (–0.1 to 0.1)	0.0 (–0.1 to 0.1)
	MOTIVATE-T2D	1.1 (0.3)	0.1 (0.0 to 0.1)	0.1 (0.0 to 0.1)		
LDL Chol, mmol/L (3%)	Active control	3.2 (1.3)	−6 (–15 to 4)	1 (–9 to 13)	4 (–9 to 19)	−10 (–21 to 4)
	MOTIVATE-T2D	3.3 (1.2)	−2 (–11 to 8)	−8 (–17 to 1)		
			% change †			
Triglycerides, mmol/L (5%)	Active control	2.0 (0.9)	−6 (–19 to 9)	0 (–15 to 16)	−1 (–18 to 21)	−4 (–21 to 18)
	MOTIVATE-T2D	2.4 (1.8)	−7 (–20 to 8)	−4 (–17 to 11)		
			% change †			
CGM TIR, % (3%)	Active control	81 (26)	−1 (–9 to 6)	−5 (–13 to 4)	3 (–7 to 12)	6 (–5 to 16)
	MOTIVATE-T2D	82 (26)	1 (–6 to 8)	1 (–6 to 8)		
CGM TBR, %	Active control	4 (8)	1 (0 to 3)	1 (0 to 2)	1 (0 to 4)	3 (1 to 14)
	MOTIVATE-T2D	4 (8)	1 (0 to 3)	2 (1 to 7)		
			ORs ‡			
CGM TAR, %	Active control	16 (26)	4 (–3 to 11)	8 (0 to 16)	−6 (–15 to 3)	−6 (–16 to 4)
	MOTIVATE-T2D	14 (27)	−2 (–8 to 5)	2 (–5 to 9)		
CGM CV, %	Active control	24 (5)	−2 (–3 to 0)	−1 (–2 to 1)	1 (0 to 3)	0 (–2 to 2)
	MOTIVATE-T2D	23 (6)	0 (–1 to 1)	−1 (–2 to 1)		
Mean glucose, mmol/L	Active control	7 (3)	6 (–1 to 14)	9 (1 to 19)	−6 (–15 to 3)	−1 (–11 to 9)
	MOTIVATE-T2D	7 (3)	0 (–7 to 6)	8 (1 to 16)		
			% change †			
Total PA, min	Active control	1484 (574)	0 (–196 to 203)	196 (7 to 385)	−28 (–287 to 231)	−105 (–343 to 126)
	MOTIVATE-T2D	1393 (490)	−28 (–217 to 161)	91 (–84 to 259)		
MVPA, min	Active control	504 (273)	56 (–21 to 126)	56 (–14 to 126)	−21 (–112 to 77)	−21 (–112 to 70)
	MOTIVATE-T2D	427 (203)	35 (–35 to 105	35 (–28 to 98)		
MVPA10+, min	Active control	105 (168)	7 (–35 to 49)	−14 (–56 to 28)	35 (–21 to 91)	14 (–35 to 63)
	MOTIVATE-T2D	49 (77)	42 (0 to 77)	0 (–35 to 35)		

All PA data is ≥4 days wear time, including ≥3 weekdays and ≥1 weekend day for ≥16-hour wear time. All CGM data is from 14-day wear time.

Where possible, minimal clinically important differences (MCIDs) have been included; HbA1c 3 mmol/mol^54 55^ which is equivalent to a 6% change from baseline in this study, weight 5% change from baseline^57^ which is equivalent to 5 kg in this study, systolic BP 5 mm Hg,^53^ diastolic BP 2 mm Hg,^64^ MAP 2 mm Hg,^64^ Chol 5% change from baseline^57^ which is equivalent to 0.3 mmol/L, HDL Chol 0.1 mmol/L,^57^ LDL Chol 0.1 mmol/L^57^ which is equivalent to 3% change from baseline in this study, triglycerides 5% change from baseline^57^ and TIR 3% change from baseline.^32^

* Within- and between-group differences adjusted for study site (UK or Canada), sex (male or female) and age (40–60 or 61–75 years).

† Log-transformed, interpret effect estimates as per cent change.

‡ Data were analysed using mixed effects binomial regression,; interpret effect estimates as odds ratiosORs. Where possible () have been included; HbA1c mmol/mol which is equivalent to a change from baseline in this study, weight change from baseline which is equivalent to kg in this study, systolic BP mmHg, diastolic BP mmHg, MAP 2mmHg, Chol change from baseline which is equivalent to mmol/L, HDL Chol mmol/L, LDL Chol mmol/L which is equivalent to change from baseline in this study, triglycerides change from baseline and TIR change from baseline.

BMI, body mass index; BP, blood pressure; CGM, continuous glucose monitor; Chol, cholesterol; CV, coefficient of variation; HDL, high density lipoprotein; LDL, low-density lipoprotein; MAP, mean arterial pressure; MOTIVATE-T2D, Mobile Health Biometrics to Enhance Exercise and Physical Activity Adherence in Type 2 Diabetes; MVPA, moderate-to-vigorous intensity PA; MVPA10+, MVPA accumulated in bout ≥10 minutes; PA, physical activity; TAR, time above range (>10.0 mmol/L); TBR, time below range (<3.9 mmol/L); TIR, time in range (3.9–10mmol/L); WC, waist circumference.

**Table 4:** Baseline and within- and between-group differences at 6- and 12- month follow-ups; questionnaires

Outcome (MCID)		Baseline, mean (SD)	Within-group difference(95% CI)*		Between-group difference(95% CI) *	
			6 months	12 months	6 months	12 months
EQ-5D-5L (0.03 to 0.05)	Active control	0.84 (0.17)	0.02 (–0.01 to 0.05)	−0.01 (–0.04 to 0.02)	0.00 (–0.04 to 0.03	0.01 (–0.03 to 0.05)
	MOTIVATE-T2D	0.83 (0.14)	0.01 (–0.01 to 0.04)	0.00 (–0.03 to 0.02)		
SF-12						
Physical outcome (3 to 5)	Active control	46 (9)	0 (–3 to 2)	−1 (–3 to 1)	0 (–3 to 3)	0 (–4 to 3)
	MOTIVATE-T2D	46 (10)	0 (–2 to 2)	−1 (–3 to 1)		
Mental (3 to 5)	Active control	49 (9)	2 (–1 to 5)	−1 (–4 to 3)	−2 (–6 to 2)	4 (0 to 8)
	MOTIVATE-T2D	46 (12)	0 (–2 to 3)	3 (0 to 6)		
DTSQs						
Derived overall score	Active control	23 (8)	4 (2 to 6)	3 (1 to 5)	1 (–2 to 4)	−1 (–4 to 2)
	MOTIVATE-T2D	21 (8)	5 (3 to 7)	2 (0 to 4)		
Burden of hyperglycaemia	Active control	3 (2)	0 (–1 to 0)	0 (–1 to 0)	0 (–1 to 0)	0 (–1 to 1)
	MOTIVATE-T2D	2 (2)	−1 (–1 to 0)	0 (–1 to 0)		
Burden of hypoglycaemia	Active control	1 (1)	0 (0 to 0)	0 (–1 to 0)	0 (–1 to 0)	0 (0 to 1)
	MOTIVATE-T2D	1 (1)	0 (–1 to 0)	0 (0 to 0)		
DTSQc†						
Derived overall score	Active control		9 (6)			
	MOTIVATE-T2D		0 (6)			
Burden of hyperglycaemia	Active control		−0 (1)			
	MOTIVATE-T2D		−0 (1)			
Burden of hypoglycaemia	Active control		−1 (1)			
	MOTIVATE-T2D		−1 (1)			

Where possible, minimal clinically important differences (MCIDs) have been included; EQ-5D-5L between 0.03 and 0.05,^65^ SF-12 physical outcome component between 3 and 5,^58^ SF-12 mental component between 3 and 5.^58^

* Within and between-group differences adjusted for centre (UK or Canada), sex (male or female) and age (40–60 or 61–75 years).Where possible () have been included; EQ-5D-5L between 0.03 and 0.05, SF-12 physical outcome component between 3 and 5, SF-12 mental component between 3 and 5

† The DTSQc was conducted at 6-month follow-up only, and the data presented are mean and SD for this time point.

DTSQc, Diabetes Treatment Satisfaction Questionnaire change version; DTSQs, Diabetes Treatment Satisfaction Questionnaire status version; MOTIVATE-T2D, Mobile Health Biometrics to Enhance Exercise and Physical Activity Adherence in Type 2 Diabetes; SF-12, 12-Item Short Form Health Survey.

### Acceptability of the MOTIVATE-T2D intervention

Qualitative interviews indicated high levels of satisfaction and acceptability of the MOTIVATE-T2D intervention in people with recently diagnosed T2D (online supplemental eTable 9). Highly valued elements of MOTIVATE-T2D included the role of the exercise specialist, where the counselling sessions and regular text messages were seen as a source of support and reassurance. The flexibility of the PA and exercise programme was also found to promote autonomy. Finally, the ability to track and monitor behaviour through the technology was viewed as an enabler. However, participants cited technical aspects of the watch and app as a challenge, highlighting the need for additional resources/training in the future.

**Figure 2:**
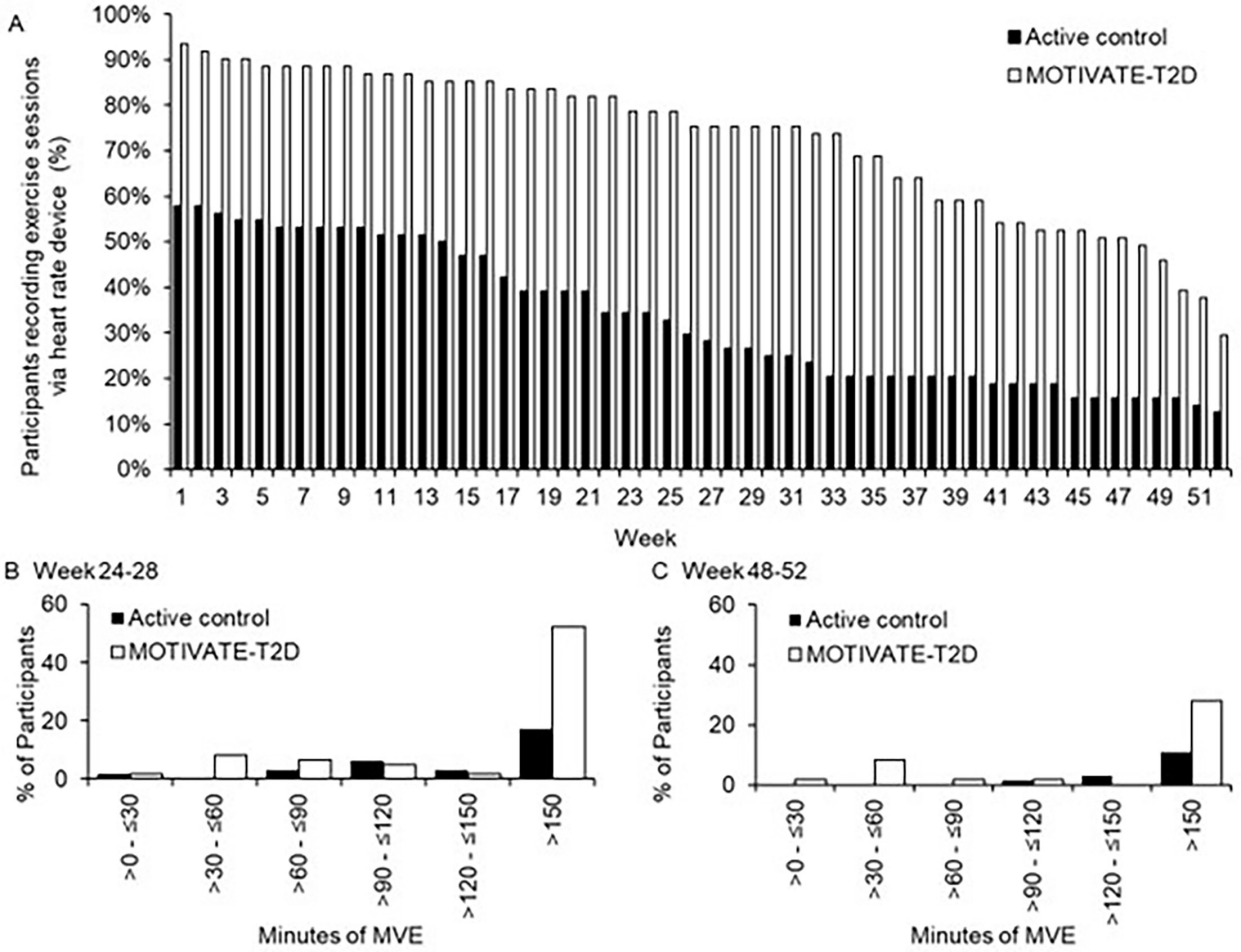
Exercise behaviour derived from optical heart rate monitoring. (A) Training drop-off by week in MOTIVATE-T2D and active control participants. (B) Proportion of participants achieving MVE duration at least once during weeks 24–28. (C) Proportion of participants achieving MVE duration at least once during weeks 48–52. MVE, moderate-to-vigorous intensity exercise; when calculating MVE, vigorous intensity exercise was multiplied by two. MOTIVATE-T2D, Mobile Health Biometrics to Enhance Exercise and Physical Activity Adherence in Type 2 Diabetes.

*Adherence:* participants in the MOTIVATE-T2D group exercised, measured via optical HR monitor, more regularly than active control during the 6month-intervention period and the 6- to 12-month follow-up (table 5). The OR of participants starting training (completed ≥1 training session) was more than 10 times higher for MOTIVATE-T2D compared with active control (OR 10.4, CI 3.4 to 32.1; MOTIVATE-T2D 93% started training, active control 58% started training). At 6 and 12 months, the OR for participants still exercising were 7 (OR 7.1, CI 3.2 to 15.7; MOTIVATE-T2D 79% still training, active control 34% still training) and 3 (OR 2.9, CI 1.2 to 7.4; MOTIVATE-T2D 30% still training, active control 13% still training) times higher for MOTIVATE-T2D compared with active control, respectively (figure 2A). At the end of the 6-onth intervention period (weeks 24–28), 52% of MOTIVATE-T2D participants completed>150 min of MVE per week at least once compared with 17% in active control (figure 2B). At the end of the 12-month follow-up period (weeks 48–52), this proportion dropped to 28% of MOTIVATE-T2D participants compared with 11% in the active control (figure 2C).

**Table 5:** Device-derived exercise behaviour during the 6-month intervention period and 6- to 12-month follow-up

	0–6 months mean (SD)	6–12 months mean (SD)	Total mean (SD)
Number of exercise sessions (n)			
Active control	1.3±1.8	0.5±1.1	0.9±1.4
MOTIVATE-T2D	3.2±2.8	1.5±2.4	2.4±2.5
Adherence to prescribed exercise (% of 78 sessions)			
Active control	47±30	–	–
MOTIVATE-T2D	83±78	–	–
Duration (min)			
Active control	77±118	30±74	54±88
MOTIVATE-T2D	182±180	88±118	135±130
Duration MVE (min)			
Active control	78±112	31±75	54±88
MOTIVATE-T2D	185±153	88±132	137±133

MVE, moderate-to-vigorous intensity exercise; when calculating MVE, vigorous intensity exercise was multiplied by two.

MOTIVATE-T2D, Mobile Health Biometrics to Enhance Exercise and Physical Activity Adherence in Type 2 Diabetes.

At 12 months, 58% of participants wore the accelerometer for 16 hours on four or more days, including one weekend day, and 71% wore the device for at least 16 hours on 1 day (online supplemental eTable 10). Wear time tended to be higher in MOTIVATE-T2D compared with the active control and in participants recruited in the UK (online supplemental eTable 12). At 6 and 12 months, within- and between-group (table 3) differences in PA outcomes were highly variable, but there was no evidence of compensatory reduction in lifestyle PA alongside increases in purposeful exercise in either group. There was evidence that wear time may have influenced between-group differences at follow-up (online supplemental eTables 15 and 16).

*Fidelity:* attendance at exercise counselling meetings was high (>80%) with little difference between groups (online supplemental eTable 19). Participant interaction with their counsellor by text message was higher in MOTIVATE-T2D (mean number of texts sent by participants (49±38) than in active control (18±14). Active control participants interacted more with the exercise videos (total video views; MOTIVATE-T2D 101, active control 135).

### Data availability

Data on likely primary outcomes, HbA1c and systolic blood pressure, were available from 95%, 78% and 74%, and 97%, 71% and 63% of participants at baseline, 6 and 12 months, respectively (online supplemental eTable 10). Availability of data for HbA1c appeared to be influenced by country, with data available from 81% of participants in the UK and 67% of participants in Canada at 12 months (online supplemental eTables 13 and 14). Data availability for secondary outcomes ranged from 58% to 74% at 12 months (online supplemental eTables 10 and 11). Wear time criteria influenced data availability for CGM, with data availability at 12 months ranging from 58% to 69% (online supplemental eTable 10). Wear time tended to be higher in MOTIVATE-T2D compared with the active control. However, wear time did not seem to influence baseline data or between-group differences (online supplemental eTables 17 and 18). Study site also appeared to influence data availability, with UK participants having higher availability of blood lipids and questionnaires, but worse availability of CGM (online supplemental eTables 12–14).

### Preliminary outcomes

**Figure 3:**
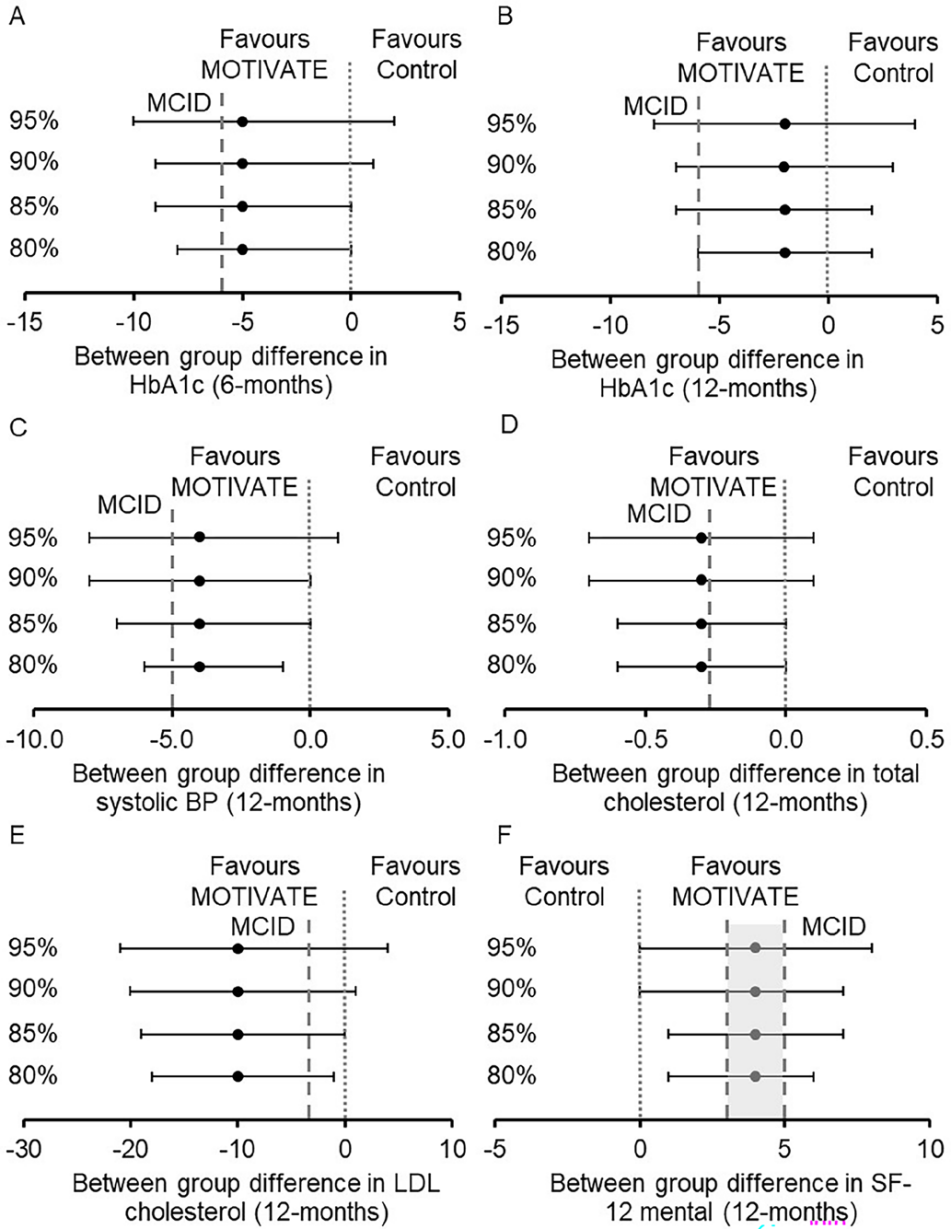
Between-group differences in outcome measures with CIs. CIs presented 95%, 90%, 85% and 80%. Minimal clinically important differences (MCIDs), indicated by ----. (A) Between-group differences in HbA1c at 6 months, MCID 3 mmol/mol^54 55^ which is equivalent to a 6% change from baseline in this study. (B) Between-group differences in HbA1c at 12 months, MCID 3 mmol/mol^54 55^ which is equivalent to a 6% change from baseline in this study. (C) Between-group differences in systolic blood pressure (BP) at 12 months, MCID 5 mm Hg.^53^ (D) Between-group differences in total cholesterol at 12 months, MCID 5% reduction.^57^ (E) Between-group differences in low-density lipoprotein (LDL) cholesterol at 12 months, MCID 0.1 mmol/L.^57^ (F) Between-group differences in the 12-item Short Form Health Survey (SF-12) mental health component score at 12 months, MCID between 3 and 5.^58^ MOTIVATE, Mobile Health Biometrics to Enhance Exercise and Physical Activity Adherence in Type 2 Diabetes.

Baseline and within- and between-group differences in exploratory clinical outcomes are shown in tables3  4. CIs and MCIDs, displayed in tables3  4, suggest plausible evidence of effect in favour of MOTIVATE-T2D for our likely primary outcomes HbA1c at 6 months and systolic blood pressure at 12 months, and other secondary outcomes at 6 and 12 months, including total cholesterol, low-density lipoprotein (LDL) cholesterol, glucose time in range and quality of life indicated by the SF-12 mental component score (figure 3).

At 6 months, changes to glucose-lowering medication were seen in 10 active control (one stopped a medication, one started a new medication, five decreased dose and three increased dose) and 13 MOTIVATE-T2D (three stopped a medication, one started a new medication, four decreased dose and five increased dose) participants. At 12 months, medication changes were seen in 12 active control (two started a new medication, four decreased dose and six increased dose) and 12 MOTIVATE-T2D (two started a new medication, three decreased dose and seven increased dose) participants. Medication changes were more common in Canadian participants (Canada 29 (46%); UK 10 (16%)).

At 6 months, four participants (UK—two and Canada—two) experienced a serious adverse event, with none of these considered related to the study processes or interventions. No serious adverse events were reported at 12 months.

### Healthcare utilisation and intervention costs

The average cost of the interventions per participant was estimated to be MOTIVATE-T2D £321.32/$C532.17 and active control £128.99/$C169.37, based on delivery of the interventions to 100 people (online supplemental eTable 20 for cost breakdown). The wider healthcare and societal utilisation for MOTIVATE-T2D and active control groups are summarised in online supplemental eTable 21.

## Discussion

The findings of this trial support the acceptability of the MOTIVATE-T2D intervention and indicate that it is feasible to recruit and retain newly diagnosed people with T2D in a randomised trial with a 12month- follow-up. MOTIVATE-T2D was well received by participants, and intervention adherence was excellent. There was evidence of higher engagement in purposeful exercise compared with the active control group and no apparent evidence of compensatory reductions in lifestyle PA. At follow-up, compared with active control, several outcomes showed a potential direction of effect in favour of MOTIVATE-T2D, including our proposed primary outcomes of HbA1c and systolic blood pressure.

The achieved recruitment and retention rates exceeded the predetermined progression criteria for the trial, with 93% of eligible people approached being randomised (criterion: >20%) and 82% of participants retained at 12 months (criterion: >80%).^24^ This recruitment rate compares well with the Diet or diet plus physical activity versus usual care in patients with newly diagnosed type 2 diabetes trial (Early-ACTID), where 98% of eligible patients with newly diagnosed T2D (5- to 8 months since diagnosis) were randomised to receive usual care or lifestyle advice.^14^ The retention rate also compares well with studies included in a systematic review of interventions using pedometers or accelerometers to promote PA in people with T2D (n=7 randomised trials, mean 78%, range 63%–98%).^13^

Early-ACTID trial, where 98% of eligible patients with newly diagnosed T2D (5- to 8 months since diagnosis) were randomised to receive usual care or lifestyle advice.^14^ The retention rate also compares well with studies included in a systematic review of interventions using pedometers or accelerometers to promote PA in people with T2D (n=7 randomised trials, mean 78%, range 63%–98%).^13^

The results from this trial suggest that the MOTIVATE-T2D approach of using biometrics from wearable technologies to support a home-delivered, personalised behavioural counselling service was promising for the promotion, uptake and adherence to purposeful exercise in people with newly diagnosed T2D. The device-derived measurements of purposeful exercise demonstrated that MOTIVATE-T2D participants were more likely to start an exercise programme and the mean weekly exercise duration was greater. Importantly, one in two MOTIVATE participants achieved the recommended 150 min of moderate-to-vigorous intensity exercise during the final month of the supported programme, compared with only one in six in the active control group. This was achieved despite both groups receiving similar support from an exercise specialist to codesign the personalised programme. It is difficult to compare the current data with previous trials where purposeful exercise has been performed unsupervised in people with T2D, as adherence data are rarely collected throughout interventions, and existing evidence has employed a variety of measurement methods.^4 10^ However, adherence, assessed as prescribed sessions attended (MOTIVATE-T2D 78%), was comparable to data reported in a systematic review of supervised exercise interventions in people with T2D (18 trials, n=523, adherence: 87±8%).^4^ A comparable intervention commissioned by health services globally, where adherence data is available, could be cardiac rehabilitation which uses a combination of supervised and unsupervised purposeful exercise to promote secondary prevention in people who have had an acute coronary event or heart failure. Adherence to MOTIVATE-T2D compares favourably to a meta-analysis (14 trials, n=8176) of cardiac rehabilitation programmes, where mean adherence (prescribed sessions attended) was 67±18.2%.^37^

During the follow-up period (6 to 12 months), participants in MOTIVATE-T2D had three times higher odds of still completing purposeful exercise versus those in the active control. However, there was a noticeable reduction in engagement with purposeful exercise during this unsupported phase following either intervention. Again, it is difficult to compare this to previous studies because of a lack of objective data on the maintenance of purposeful exercise in unsupervised trials. However, studies measuring PA have shown that maintaining behaviour change after the conclusion of an intervention is challenging.^38^ Data from our qualitative evaluation suggest that extending the text message feedback period may be a simple and cost-effective way of supporting participants to maintain changes in behaviour. As such, future iterations of the intervention should explore the feasibility and cost implications of such a refinement. Future iterations could also look to introduce more social interactions to the intervention, as social connectedness has been shown to be an important determinant of long-term adherence.^39 40^ Such interactions were difficult to incorporate into the current trial due to COVID-19 restrictions and small numbers limiting the use of online forums.

As per the study design, the active control group also completed a complex intervention containing a range of behaviour change techniques that have previously been associated with effective PA interventions, including goal setting, action planning, implementing graded tasks and self-monitoring of behaviour.^41^,^45^ However, our findings suggest the addition of biofeedback to support a complex behavioural intervention was an effective strategy to partner with behaviour change. In particular, the provision of HR monitoring to guide participants’ purposeful exercise in real time and facilitate personalised feedback from exercise specialists appeared to be an important strategy within the MOTIVATE-T2D intervention. Potential mechanisms of action used throughout the MOTIVATE-T2D intervention will be explored more in a complementary paper.

Alongside the measurement of purposeful exercise, lifestyle PA was assessed by an accelerometer. This combined measurement strategy reflected the two-step intervention approach where engagement in purposeful exercise was encouraged alongside unstructured lifestyle-based PA. It was difficult to draw conclusions from the PA data due to the small sample size and large variability. However, increased engagement in purposeful exercise did not seem to lead to a compensatory reduction in lifestyle-based PA, which has previously been cited as a concern during supervised exercise interventions.^46 47^ The difficulty interpreting the PA data potentially reflects the challenges of using accelerometers to assess an intervention which encourages participation in purposeful exercise alongside ambulatory PA. Both the active control and MOTIVATE-T2D interventions encouraged participants to take part in types/modes of exercise whose intensity may not have been accurately captured by the accelerometer (eg, cycling or resistance exercise).^19 46^ Future studies could look to include cardiorespiratory fitness as an index of intervention effectiveness alongside lifestyle-based PA and engagement in purposeful exercise. Sustained increases in cardiorespiratory fitness following the Italian Diabetes Exercise Study 2, which targeted reallocation of sedentary time with light-intensity activity and purposeful MVPA in people with T2D, were predictive of improvements in HbA1c and coronary heart disease risk, independent of changes in MVPA or sedentary time.^48^

Due to the association of HbA1c and systolic blood pressure with diabetes complications and mortality,^49^,^51^ the goal for clinical management of T2D is to achieve and maintain tight control of HbA1c and systolic blood pressure.^52^ Therefore, HbA1c and systolic blood pressure are likely to be primary outcomes in a future RCT. Lee *et al*^35^ and Bell *et al*^34^ suggest using CIs and the MCID to interpret feasibility trials. Using this approach, there was evidence that a clinically important difference in HbA1c at 6 months and systolic blood pressure at 12 months between MOTIVATE-T2D and active control was plausible. Studies report a 5 mm Hg reduction in systolic blood pressure as the MCID,^53^ and the 95% CIs for systolic blood pressure in this study included 5 mm Hg. Studies also suggest a difference of 3 mmol/mol would represent a clinically important difference in HbA1c,^54 55^ and the −5% between-group difference at 6 months is equivalent to ~3 mmol/mol. However, the between-group difference for HbA1c was not maintained at 12 months. As discussed above, future iterations of the intervention should look to improve the maintenance of purposeful exercise with the aim of sustaining long-term improvements in HbA1c. It is difficult to compare the between-group differences in HbA1c and systolic blood pressure to previous research as most RCTs use usual care control groups rather than the contact-matched active control group in the current study. However, meta-analysis of unsupervised behavioural interventions suggested that they were not associated with changes in HbA1c unless combined with dietary advice.^4^ A similar meta-analysis of unsupervised behavioural interventions in people with T2D on systolic blood pressure suggested a small (weighted mean difference of 3 mm Hg, 95% CI −5 to −1) but significant effect.^56^ As well as the encouraging data for HbA1c and systolic blood pressure, there was evidence that a clinically important difference may be plausible for a number of secondary outcomes, including total cholesterol and LDL cholesterol at 12 months^57^ and quality of life at 12 months, indicated by the SF-12 mental component score.^58^ The UK prospective diabetes study showed the importance of dyslipidaemia for cardiovascular disease risk in people with T2D.^59^ As such, potential improvements in these secondary outcomes are highly relevant for people with T2D. As discussed above, the active control group received a complex intervention, and it is plausible that between-group differences would have been larger if compared with a usual care control group, increasing the likelihood of clinically important effects in the real world.

### Implications for planning a future trial

Based on HbA1c as the primary outcome, a full trial comparing MOTIVATE-T2D versus active control would require the recruitment of 586 participants with recently diagnosed T2D. This estimate is based on detecting a minimum clinically important difference of 3 mmol/mol,^54 55^ an SD at baseline of 10 mmol/mol (as seen in this feasibility trial) and an assumed attrition rate of 20% (as seen in this feasibility trial), at 90% power and a two-tailed 5% α level.

Due to restrictions and uncertainties caused by the COVID-19 pandemic, the trial used a decentralised design. Our experience was that the decentralised design could positively impact trial feasibility, as recruitment was not limited by geographic constraints. As suggested previously,^60^ the decentralised design may also have provided an opportunity for greater diversity in our trial population. Compared with Early-ACTID, which also recruited people with newly diagnosed T2D in the UK, the current trial recruited a more ethnically diverse population.^14^ Participant interviews suggested that the decentralised approach was broadly acceptable, although future iterations may need to consider how participants are supported with taking blood samples. Considerations should also be made for differences between the UK and Canada in research infrastructure. During study planning, there were no services that could process capillary blood samples in Canada. This led to the approach of shipping samples to the UK for analysis. We believe this additional step may have been responsible for the reduced HbA1c and blood lipid data availability in Canada. Although data availability for outcome measures was good, the centralised Early-ACTID trial collected primary outcome data (HbA1c) from 98% of participants at 12 months. Simple refinements to the study procedures could be made to collect more complete and meaningful data. This could be achieved by providing greater financial incentives.^61^ Changes to survey formatting and better use of data validation filters to reduce incorrectly entered measures, which accounted for approximately one-third of the missing questionnaire and anthropometric data. Finally, the use of next-generation PA monitors that collect accelerometer data in real-time could increase compliance with wear time criteria.^62^

This study has some limitations. First, the study was not designed or powered to definitively assess the efficacy of MOTIVATE-T2D in people with recently diagnosed T2D. Second, there was evidence of imbalances between intervention and active control groups in their demographic characteristics. Third, participant and researcher blinding was not possible because of the nature of the intervention. Fourth, it is not known if active control participants wore the blinded HR monitor for all purposeful exercise sessions. Therefore, device-derived purposeful exercise metrics may be underestimated in the active control group. Finally, the study did not employ a strict target-driven approach to the regulation of glucose-lowering medication, which may have influenced outcomes.^63^ Given these limitations and the feasibility design of this trial, our findings should be considered preliminary, and encouraging trends require confirmation in a larger, adequately powered RCT.

In summary, the findings of this feasibility trial indicate that the MOTIVATE-T2D intervention is feasible and acceptable, with promising effects on adherence to purposeful exercise. This feasibility study will inform the funding application for a fully powered RCT to assess the clinical effectiveness and cost-effectiveness of the MOTIVATE-T2D intervention in people with recently diagnosed T2D.

## Supplementary material

10.1136/bmjopen-2024-092260 online supplemental file 1

## Data Availability

Data are available upon reasonable request.
