# Supplementary material for: Mobile Health Biometrics to Enhance Exercise and Physical Activity Adherence in Type 2 Diabetes (MOTIVATE-T2D): a decentralised feasibility randomised controlled trial delivered across the UK and Canada
Source: BMJ Open. 2025 Mar 26;15(3):e092260. doi: 10.1136/bmjopen-2024-092260 (PMC12004491; doi:10.1136/bmjopen-2024-092260)
Supplement: online supplemental file 1 [file bmjopen-15-3-s001.docx]

**Online supplementary eFigures and eTables**

**
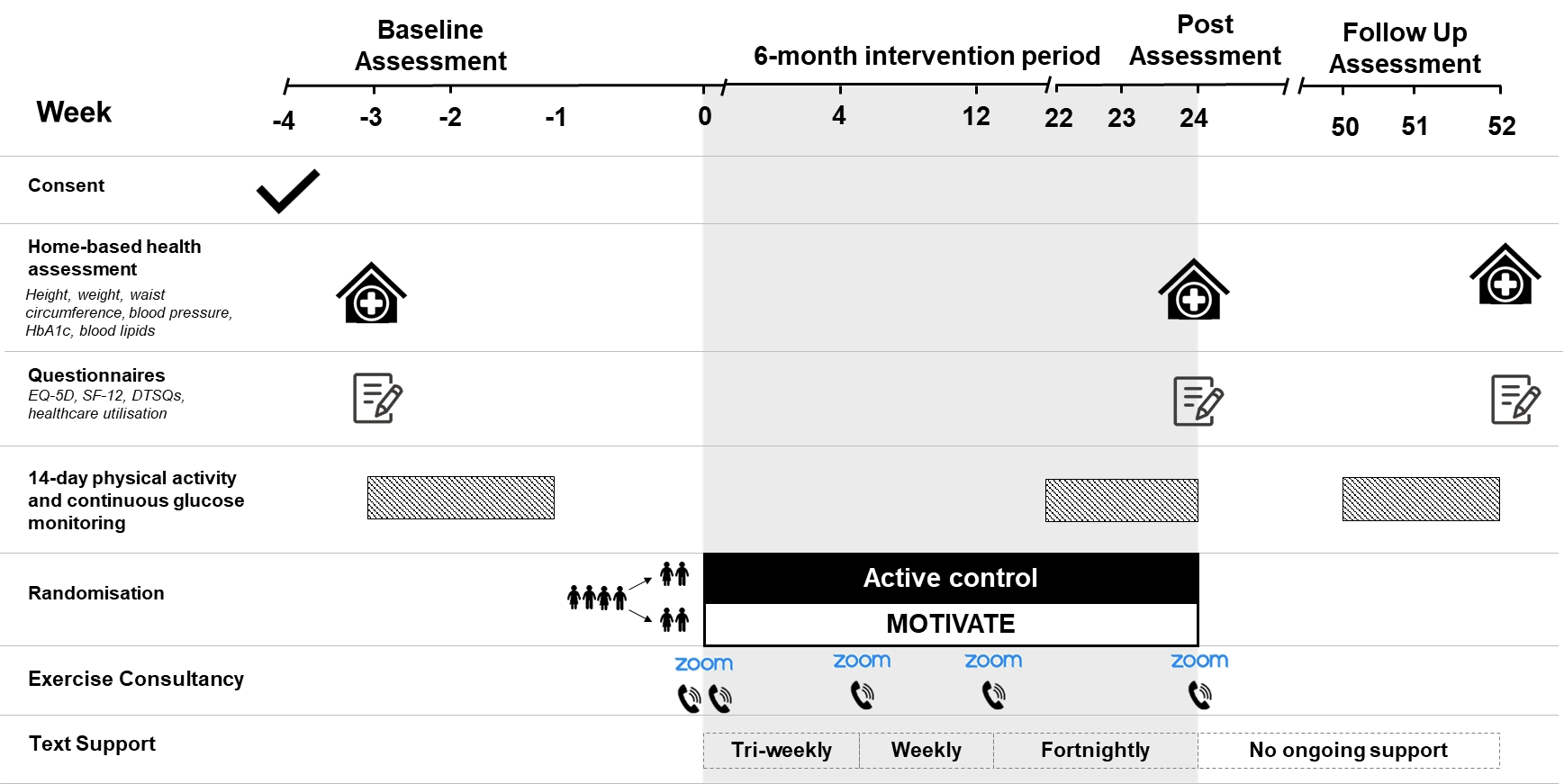
**

eFigure 1. Schematic of the study timeline.


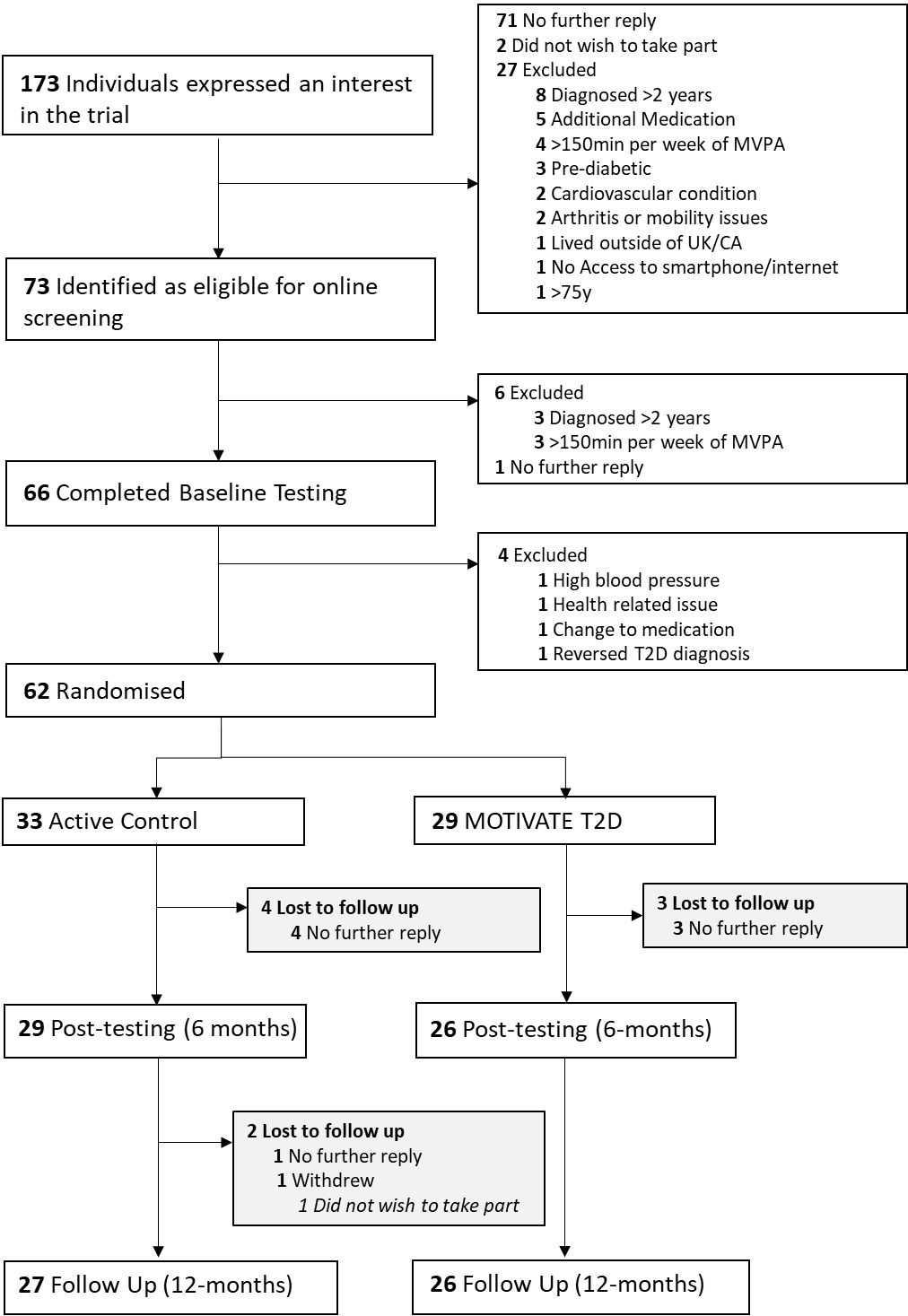


eFigure 2. UK only Consolidated Standards of Reporting Trials (CONSORT) flow chart.


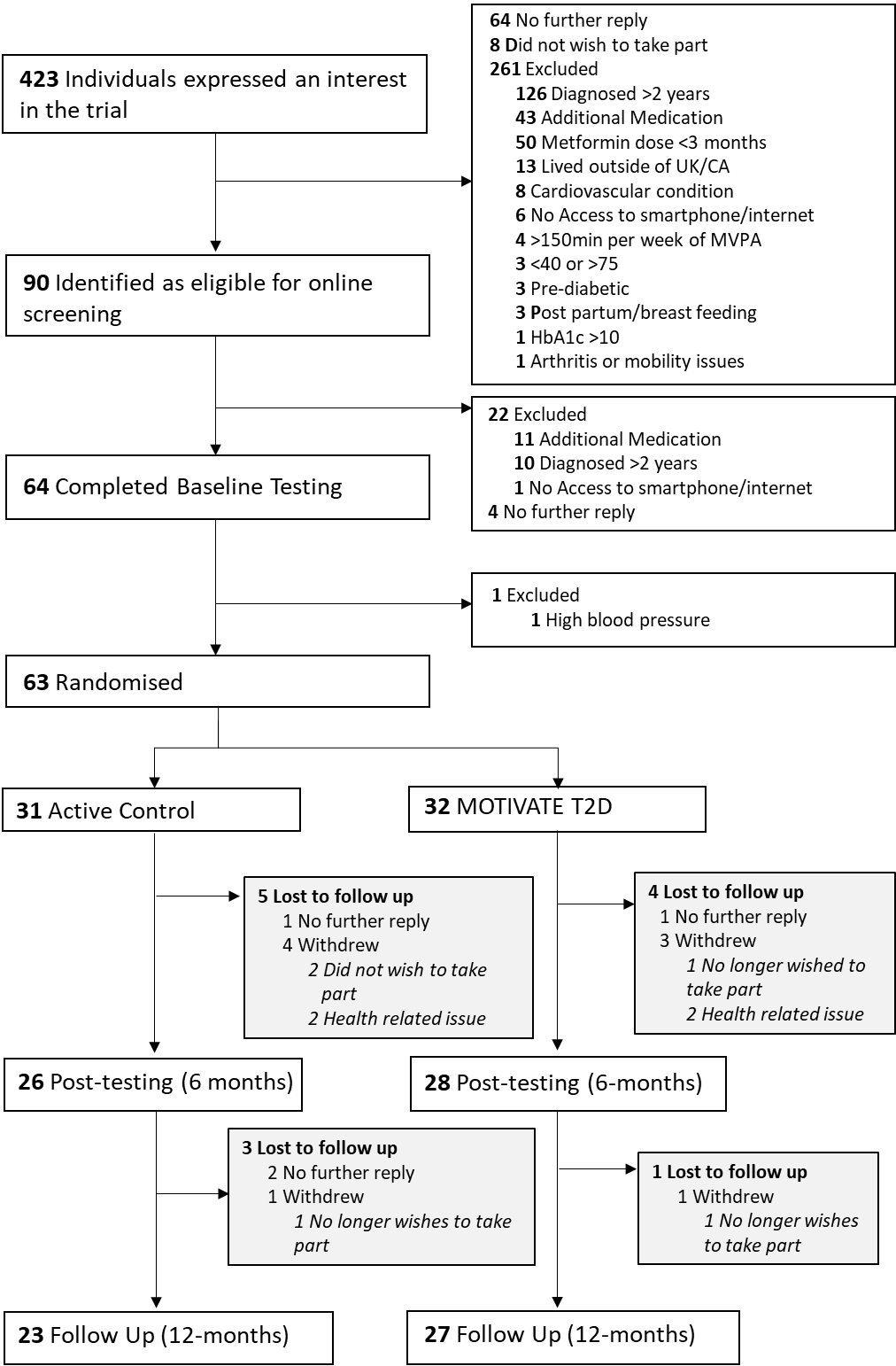


eFigure 3. Canada only Consolidated Standards of Reporting Trials (CONSORT) flow chart.

eTable 1. Influence of recruitment strategy on expressions of interest, participants randomised and recruitment rate

| Recruitment strategy | Expressions of interest, N (% of expressions of interest n=596) | Excluded, N (% of expression of interest for strategy) | Did not reply, N (% of expression of interest for strategy) | Randomised, N (% of those randomised n=125) | Success rate, % of expression of interest for strategy randomised |
| --- | --- | --- | --- | --- | --- |
| GP database searches | 94 (16) | 13 (14) | 37 (39) | 44 (35) | 47 |
| Third party recruitment Services | 326 (55) | 239 (73) | 49 (15) | 37 (30) | 11 |
| Local media articles/ classifieds | 83 (14) | 54 (65) | 18 (22) | 11 (9) | 13 |
| Diabetes education sessions | 10 (2) | 0 (0) | 0 (0) | 10 (8) | 100 |
| Referral from Friend | 12 (2) | 2 (17) | 0 (0) | 10 (8) | 83 |
| Adverts in clinical settings | 24 (4) | 15 (63) | 3 (2) | 6 (5) | 25 |
| Consent to Contact Database | 9 (2) | 0 (0) | 4 (44) | 5 (4) | 56 |
| Study Website | 10 (2) | 7 (70) | 2 (20) | 1 (1) | 10 |
| Unknown | 15 (3) | 0 (0) | 15 (100) | 0 (0) | 0 |
| Advert on Diabetes Canada social media | 13 (2) | 0 (0) | 12 (100) | 1 (1) | 8 |

eTable 2. Influence of recruitment source on participant demographics

| Recruitment strategy, N= participants recruited | Male,  N (%) | >60,  N (%) | White,  N (%) | University education, N (%) | Full time employment, N (%) |
| --- | --- | --- | --- | --- | --- |
| GP database searches, N=44 | 23 (52) | 15 (34) | 40 (91) | 17 (17) | 26 (59) |
| Third party recruitment Services, N=37 | 22 (59) | 9 (24) | 25 (68) | 19 (51) | 26 (70) |
| Local media articles, N=11 | 6 (55) | 8 (73) | 11 (100) | 5 (45) | 4 (36) |
| Diabetes education sessions, N=10 | 7 (70) | 1 (10) | 8 (80) | 4 (40) | 5 (50) |
| Referral from Friend, N=10 | 4 (40) | 2 (20) | 7 (70) | 3 (30) | 6 (60) |
| Adverts in clinical settings, N=6 | 1 (17) | 1 (17) | 3 (50) | 5 (83) | 3 (50) |
| Consent to Contact Database, N=5 | 2 (40) | 1 (20) | 5 (100) | 2 (40) | 3 (60) |
| Study Website, N=1 | 0 | 0 | 1 (100) | 1 (100) | 1 (100) |
| Advert on Diabetes Canada social media, N=1 | 0 | 0 | 1 (100) | 0 | 1 (100) |

eTable 3. Participant satisfaction with their involvement in the trial

| **The following are verbatim quotes regarding the research process:** |
| --- |
| The way it works is pretty good, the technology side of it, Zoom / Teams…it works very well so the communication side is good.” (Participant 17-UK) |
| [If the trial were to be repeated in a clinic setting] “for me personally I would find that hard to get to because I don’t drive now so this part of it being able to do in your own home I think is really good.” (Participant 20-UK) |
| “All the instructions were clear, the feedback I had to give on my results, what I had to do with the equipment, returning and stuff, it was adequate really, more than adequate.”  “it was all prepaid envelope, I just took it to the main post office sealed, sent off, no problem, we have a post office very close by so that was just a little job while you’re out shopping.” (Participant 52-UK) |
| “I don't know that I had many challenges, because it was provided in such a seamless, customized format, that there really, I wouldn't say I was challenged in any way.”  “it's been such a pleasant experience that I'm more willing to engage as a research participant again.” (Participant 02-CA) |
| “I’m a bit pen and paper person rather than technology but I think everything was really well, really explained so well and so easy to follow and you had that back up, that support if you needed it.”  “the testing booklet, that was really, really helpful and [the researcher] also gave me a link to a website that actually showed like videos of how to do the finger prick test and also how to attach the blood glucose monitor…it was very easy to follow” (Participant 02-UK) |
| “everything was well laid out. I really liked the detail on the instructions on what to use and how to use it and I particularly thought it was a good personal touch that the actual research instigator was the guinea pig so to speak with all the pictures so yeah it’s a personal touch but also it kind of entrusts you that they’ve gone through it, they’ve had to endure it so yeah everything worked fine that side.” (Participant 01-UK) |
| “I found that the questionnaires that I had to fill in irritating because they didn’t see coherent, they weren’t set out to say we’re looking at this, tell us about that, they didn’t ask questions which would allow me then to go on and make a sensible answer. I didn’t find their terms particularly well.” (Participant 27-UK) |
| “I’m not comfortable with needles. When I opened the box and saw that there was a needle in the middle, I started to panic, thinking that’s going to hurt… I had to look at the video. I was a bit nervous, but the little needle didn’t hurt at all” |
| “The amount of blood that they're looking for is significant from just a basically a finger prick, which is what the little device does. And it was hard to get. I never got enough blood into the vial.” |

eTable 4. Baseline medications

| N (%) | Total | Active control | MOTIVATE-T2D |
| --- | --- | --- | --- |
| Oral hypoglycaemic agents |  |  |  |
| Metformin | 69 (55) | 37 (58) | 32 (52) |
| Antihypertensive agents | 78 (62) | 40 (63) | 40 (62) |
| Renin-angiotensin agent | 35 (28) | 21 (33) | 14 (23) |
| Thiazide diuretic | 12 (10) | 4 (6) | 8 (13) |
| β blocker | 11 (9) | 5 (8) | 6 (10) |
| Calcium-channel blocker | 19 (15) | 10 (16) | 9 (15) |
| Other | 1 (1) | 0 (0) | 1 (2) |
| Lipid-lowering drugs | 50 (40) | 25 (39) | 25 (41) |
| Statin | 49 (39) | 24 (38) | 25 (41) |
| Selective cholesterol-absorption inhibitor | 1 (1) | 1 (2) | 0 (0) |

eTable 5. Baseline demographic characteristics: UK

|  | Total | Active control | MOTIVATE-T2D |
| --- | --- | --- | --- |
| N | 62 | 33 | 29 |
| Age, years, mean (SD) | 55 (9) | 56 (9) | 56 (8) |
| Female, N (%) | 30 (48) | 16 (48) | 14 (48) |
| Male, N (%) | 32 (52) | 17 (52) | 15 (52) |
| Duration of T2D, months, mean (SD) | 13 (6) | 12 (6) | 12 (6) |
| Ethnicity​, N (%) |  |  |  |
| White​ | 54 (87) | 30 (91) | 24 (83) |
| African or Caribbean | 3 (5) | 2 (6) | 1 (3) |
| Asian​ | 2 (3) | 1 (3) | 1 (3) |
| Other or mixed | 3 (5) | 0 (0) | 3 (10) |
| Marital status, living arrangements, N (%) |  |  |  |
| Married, living with spouse | 41 (66) | 20 (61) | 21 (72) |
| Married, living arrangement unknown | 1 (2) | 0 (0) | 1 (3) |
| Single, living alone | 9 (15) | 7 (21) | 2 (7) |
| Single, living with others | 2 (3) | 2 (6) | 0 (0) |
| Separated, living alone | 5 (8) | 2 (6) | 3 (10) |
| Separated, living with spouse/partner | 1 (2) | 0 (0) | 1 (3) |
| Widowed, living alone | 2 (3) | 1 (3) | 1 (3) |
| Rather not say, living with spouse/ partner | 1 (2) | 1 (3) | 0 (0) |
| Educational Attainment, N (%) |  |  |  |
| Secondary | 13 (21) | 8 (24) | 5 (17) |
| Further | 24 (39) | 9 (27) | 15 (52) |
| Higher | 25 (40) | 16 (48) | 9 (31) |
| Employment Situation, N (%) |  |  |  |
| Full Time | 33 (53) | 16 (48) | 17 (59) |
| Part Time | 6(10) | 3 (9) | 3 (10) |
| Retired | 11 (18) | 7 (21) | 4 (14) |
| Student | 1 (2) | 0 (0) | 1 (3) |
| Volunteer | 0 (0) | 0 (0) | 0 (0) |
| Stay at Home | 1 (2) | 1 (3) | 0 (0) |
| Unable: Care | 1 (2) | 0 (0) | 1 (3) |
| Unable: Health | 8 (13) | 5 (13) | 3 (10) |
| Unemployed | 1 (2) | 1 (2) | 0 (0) |

eTable 6. Baseline medications: UK

| N (%) | Total | Active control | MOTIVATE-T2D |
| --- | --- | --- | --- |
| Oral hypoglycaemic agents |  |  |  |
| Metformin | 26 (42) | 17 (52) | 9 (31) |
| Antihypertensive agents | 50 (81) | 29 (88) | 21 (72) |
| Renin-angiotensin agent | 23 (37) | 16 (48) | 7 (24) |
| Thiazide diuretic | 5 (8) | 2 (6) | 3 (10) |
| β blocker | 7 (11) | 4 (12) | 3 (10) |
| Calcium-channel blocker | 14 (23) | 7 (21) | 7 (24) |
| Other | 1 (2) | 0 (0) | 1 (3) |
| Lipid-lowering drugs | 30 (48) | 17 (52) | 13 (45) |
| Statin | 30 (48) | 17 (52) | 13 (45) |
| Selective cholesterol-absorption inhibitor | 0 (0) | 0 (0) | 0 (0) |

eTable 7. Baseline demographic characteristics: Canada

|  | Total | Active control | MOTIVATE-T2D |
| --- | --- | --- | --- |
| N | 63 | 31 | 32 |
| Age, years, mean (SD) | 54 (9) | 53 (8) | 53 (9) |
| Female, N (%) | 30 (48) | 15 (48) | 15 (47) |
| Male, N (%) | 33 (53) | 16 (52) | 17 (53) |
| Duration of T2D, months, mean (SD) | 14 (7) | 14 (7) | 14 (7) |
| Ethnicity​, N (%) |  |  |  |
| White​ | 47 (76) | 22 (71) | 25 (78) |
| African or Caribbean | 2 (3) | 0 (0) | 2 (6) |
| Asian​ | 12 (23) | 7 (23) | 5 (16) |
| Other or mixed | 2 (3) | 2 (6) | 0 (0) |
| Marital status, living arrangements, N (%) |  |  |  |
| Married, living with spouse | 49 (78) | 24 (77) | 25 (78) |
| Married, living alone | 1 (2) | 0 (0) | 1 (3) |
| Single, living alone | 6 (10) | 3 (10) | 3 (9) |
| Single, living with child | 1 (2) | 0 (0) | 1 (3) |
| Single, living arrangement unknown | 1 (2) | 1 (3) | 0 (0) |
| Separated, living alone | 4 (6) | 3 (10) | 1 (3) |
| Widowed, living with spouse/partner | 1 (2) | 0 (0) | 1 (3) |
| Educational Attainment, N (%) |  |  |  |
| Secondary | 6 (10) | 3 (10) | 3 (9) |
| Further | 26 (41) | 14 (45) | 12 (38) |
| Higher | 31 (49) | 14 (45) | 17 (53) |
| Employment Situation, N (%) |  |  |  |
| Full Time | 42 (68) | 24 (73) | 18 (62) |
| Part Time | 5 (8) | 1 (3) | 4 (14) |
| Retired | 10 (16) | 4 (120 | 6 (21) |
| Student | 1 (2) | 1 (3) | 0 (0) |
| Volunteer | 2 (3) | 1 (3) | 1 (3) |
| Stay at Home | 0 (0) | 0 (0) | 0 (0) |
| Unable: Care | 0 (0) | 0( 0) | 0 (0) |
| Unable: Health | 1 (2) | 0 (0) | 1 (3) |
| Unemployed | 2 (3) | 0 (0) | 2 (7) |

eTable 8. Baseline medications: Canada

| N (%) | Total | Active control | MOTIVATE-T2D |
| --- | --- | --- | --- |
| Oral hypoglycaemic agents |  |  |  |
| Metformin | 43 (68) | 20 (65) | 23 (72) |
| Antihypertensive agents | 28 (44) | 11 (35) | 17 (53) |
| Renin-angiotensin agent | 12 (19) | 5 (16) | 7 (22) |
| Thiazide diuretic | 7 (11) | 2 (6) | 5 (16) |
| β blocker | 4 (6) | 1 (3) | 3 (9) |
| Calcium-channel blocker | 5 (8) | 3 (10) | 2 (6) |
| Other | 0 (0) | 0 (0) | 0 (0) |
| Lipid-lowering drugs | 20 (32) | 8 (26) | 12 (38) |
| Statin | 19 (30) | 7 (23) | 12 (38) |
| Selective cholesterol-absorption inhibitor | 1 (2) | 1 (3) | 0 (0) |

eTable 9. Participant acceptability of MOTIVATE-T2D intervention

| **The following are verbatim quotes of the positive experiences of MOTIVATE-T2D from participants** |
| --- |
| **Support from the exercise counsellor (counselling sessions and text messages** |
| “I think that the interactivity with someone you get to know and trust and feel a somewhat, albeit virtual connection to has been … I'll say the backbone of the program. If somebody emailed this to me, I don't think I would have taken it to heart as much as having a personal connection.” |
| “The motivational texts that came in once a week, and then every other week, I found to be very helpful. …it was personalized, which I really liked. And then I would respond back” |
| **Flexibility of the physical activity program** |
| “The goal setting process was very realistic and directed at me, I didn't feel pressured to do more than I wanted to, rather it just kind of supported what I wanted to get out of it.” |
| “One of the things that helped me a fair bit was the flexibility I had with the exercise because I developed some of my own exercise routines…I have a pool in the backyard, so I developed some routines there for exercising in the water.” |
| **Tracking and monitoring behaviour** |
| “It made me more aware…I saw the numbers and I knew if I was doing well, …then if it wasn't going in the right direction, I had to do a little bit more.” |
| “I think it's just part of my life now…I'm always using it, recording stuff… looking at how I'm doing… for the number of steps I make. I always have targets…so it does motivate me to make sure that I meet my goals each day.” |
| **Technical aspects of the watch and mobile app** |
| “It was great when K was running things for me, but a bit more instruction on how to use the watch would have been useful when I got to do things myself.” |
| "it's certainly easier to program, to plan your sessions through the computer as opposed to on the app. … if there was some way of making that better on the app." |

eTable 10. Data availability: HbA1c, anthropometrics, blood pressure, device derived physical activity and continous glucose monitoring

| All data Control/MOTIVATE-T2D | Baseline, N (%) | 6-month, N (%) | 12-month, N (%) |
| --- | --- | --- | --- |
| Drop-out | - | 109 (87) | 103 (82) |
| HbA1c | 119 (95)  63 (98) / 56 (92) | 98 (78)  48 (75) / 50 (82) | 92 (74)  44 (69) / 48 (79) |
| Weight | 125 (100) | 87 (70)  42 (66) / 46 (75) | 80 (64)  39 (61) / 41 (67) |
| Waist circumference | 125 (100) | 87 (70)  42 (65) / 46 (75) | 77 (62)  38 (59) / 39 (64) |
| Systolic blood pressure | 121 (97)  63 (98) / 58 (95) | 89 (71)  43 (67) / 46 (75) | 79 (63)  38 (59) / 41 (67) |
| Diastolic blood pressure | 122 (98)  63 (98) / 59 (97) | 89 (71)  43 (67) / 46 (75) | 78 (62)  38 (59) / 40 (66) |
| Total Cholesterol | 85 (68)  43 (67) / 41 (67) | 63 (50)  33 (52) / 30 (49) | 64 (51) 30 (47) / 34 (56) |
| HDL Cholesterol | 93 (74)  48 (75) 45 (74) | 76 (61)  35 (55) / 41 (67) | 74 (59)  35 (55) / 39 (64) |
| LDL Cholesterol | 81 (65)  44 (69) / 37 (61) | 68 (54)  33 (52) / 35 (57) | 62 (50)  28 (44) / (34 (56) |
| Triglycerides | 83 (66)  43 (41) / 40 (66) | 67 (54)  33 (52) / 34 (56) | 63 (50)  29 (45) / 34 (56) |
| Device derived PA: Met wear time criteria | | | |
| 4-day (3 WD, 1WE) | 105 (84)  54 (85) / 51 (84) | 61 (49)  28 (48) / 33 (54) | 73 (58)  32 (50) / 41 (67) |
| 3-day (2WD, 1WE) | 105  54 (84) / 51 (84) | 61 (49)  28 (44) / 33 (54) | 76 (61)  34 (53) / 42 (69) |
| 3-day (any day) | 112 (90)  57 (89) / 55 (90) | 65 (52)  30 (47) / 35 (57) | 80 (64)  38 (59) / 42 (69) |
| 1-day | 116 (93)  60 (94) / 56 (92) | 71 (57)  34 (53) / 37 (61) | 89 (71)  42 (66) / 47 (77) |
| CGM: Met wear time criteria | | | |
| 14-Day | 95 (76)  46 (72) / 49 (80) | 90 (72)  43 (67) / 47 (77) | 72 (58)  30 (47) / 42 (69) |
| 10-Day | 105 (84)  52 (81) / 53 (87) | 93 (74)  45 (70) / 48 (79) | 75 (60)  30 (47) / 45 (74) |
| 7-Day | 107 (86)  54 (58) / 53 (87) | 97 (78)  49 (77) / 48 (79) | 86 (69)  37 (58) / 49 (80) |

HDL, high density lipoprotein; LDL, low density lipoprotein; PA, physical activity; CGM, continous glucose monitor; WD, week day; WE, weekend day; All PA data is ≥16h wear time; 14-day wear time for CGM achieved if ≥70% of data available; 10- and 7-day wear time for CGM achieved if ≥80% of data available

eTable 11. Data availability: Questionnaires

| All data Control/MOTIVATE-T2D | Baseline, N (%) | 6-month, N (%) | 12-month, N (%) |
| --- | --- | --- | --- |
|  |  |  |  |
| Drop-out | - | 109 (87) | 103 (82) |
| EQ-5D-5L | 125 (100) | 94 (75) 44 (69) / 51 (84) | 88 (70) 41 (64) / 47 (77) |
| SF12 | 118 (94) 63 (98) / 55 (90) | 88 (70) 41 (64) / 47 (77) | 82 (66) 39 (61) / 43 (70) |
| DTSQs | 125 (100) | 94 (75) 44 (69) / 50 (82) | 86 (69) 41 (64) / 45 (74) |
| DTSQc | - | 92 (74) 43 (67) / 49 (80) | - |
| Healthcare usage | 121 (97) 64 (100) / 57 (93) | 93 (74) 44 (69) / 49 (80) | 86 (69) 40 (63) / 46 (75) |

VAS, visual analogue scale; SF12, SF-12 Health Survey; BREQ-2, Behavioural Regulation in Exercise Questionnaire version 2; DTSQs, Diabetes Treatment Satisfaction Questionnaire status version, DTSQc, Diabetes Treatment Satisfaction Questionnaire change version. DTSQc was only taken at 6-months post.

eTable 12. UK and Canada data availability: Device derived physical activity and continous glucose monitoring

|  | UK | | | Canada | | |
| --- | --- | --- | --- | --- | --- | --- |
|  | Baseline, N (%) | 6-month, N (%) | 12-month, N (%) | Baseline, N (%) | 6-month, N (%) | 12-month, N (%) |
| Drop-out | - | 55 (89) | 53 (85) | - | 54 (86) | 50 (79) |
| PA: Met wear time | | | | | | |
| 4-day (3 WD, 1WE) | 54 (87) | 17 (27) | 40 (65) | 51 (82) | 44 (70) | 33 (52) |
| 3-day (2WD, 1WE) | 54 (87) | 17 (27) | 40 (65) | 51 (81) | 44 (70) | 36 (57) |
| 3-day (any day) | 57 (92) | 19 (31) | 41 (66) | 55 (87) | 46 (73) | 39 (62) |
| 1-day | 59 (95) | 21 (34) | 46 (74) | 57 (90) | 50 (79) | 43 (68) |
| CGM: Met wear time | |  |  |  |  |  |
| 14-Day | 43 (69) | 45 (73) | 30 (48) | 53 (83) | 45 (71) | 42 (67) |
| 10-Day | 48 (77) | 48 (77) | 32 (52) | 58 (92) | 46 (73) | 43 (68) |
| 7-Day | 49 (79) | 51 (82) | 39 (63) | 59 (94) | 47 (75) | 47 (75) |

PA, physical activity; CGM, continous glucose monitor; WD, week day; WE, weekend day; All PA data is ≥16h wear time; 14-day wear time for CGM achieved if ≥70% of data available; 10- and 14-day wear time for CGM achieved if ≥80% of data available

eTable 13. UK and Canada data availability: HbA1c, anthropometrics, blood pressure and blood lipids

|  | UK | | | Canada | | |
| --- | --- | --- | --- | --- | --- | --- |
|  | Baseline, N (%) | 6-month, N (%) | 12-month, N (%) | Baseline, N (%) | 6-month, N (%) | 12-month, N (%) |
| Drop-out | - | 55 (89) | 53 (85) | - | 54 (86) | 50 (79) |
| HbA1c | 62 (100) | 50 (81) | 50 (81) | 57 (90) | 48 (76) | 42 (67) |
| Weight | 62 (100) | 41 (66) | 42 (68) | 63 (100) | 46 (73) | 38 (60) |
| Waist circumference | 62 (100) | 42 (68) | 39 (63) | 63 (100) | 45 (71) | 38 (60) |
| Systolic blood pressure | 60 (97) | 43 (69) | 40 (65) | 61 (97) | 46 (73) | 39 (62) |
| Diastolic blood pressure | 60 (97) | 43 (69) | 40 (65) | 62 (98) | 46 (73) | 38 (60) |
| Total Cholesterol | 43 (69) | 33 (53) | 37 (60) | 41 (65) | 30 (48) | 27 (43) |
| HDL Cholesterol | 47 (76) | 36 (58) | 43 (69) | 46 (73) | 40 (63) | 31 (49) |
| LDL Cholesterol | 42 (68) | 33 (53) | 36 (58) | 39 (62) | 35 (56) | 26 (41) |
| Triglycerides | 43 (69) | 32 (52) | 36 (58) | 40 (63) | 35 (56) | 27 (43) |

HDL, high density lipoprotein; LDL, low density lipoprotein

eTable 14. UK and Canada data availability: Questionnaires

|  | UK | | | Canada | | |
| --- | --- | --- | --- | --- | --- | --- |
|  | Baseline, N (%) | 6-month, N (%) | 12-month, N (%) | Baseline, N (%) | 6-month, N (%) | 12-month, N (%) |
| Drop-out | - | 55 (89) | 53 (85) | - | 54 (86) | 50 (79) |
| EQ-5D-5L | 62 (100) | 48 (77) | 49 (79) | 63 (100) | 46 (73) | 39 (62) |
| SF12 | 57 (92) | 45 (73) | 45 (73) | 61 (97) | 43 (68) | 37 (59) |
| DTSQs | 62 (100) | 48 (77) | 49 (79) | 63 (100) | 46 (73) | 37 (59) |
| DTSQc | - | 46 (74) | - | - | 46 (73) | - |
| Healthcare usage | 58 (94) | 48 (77) | 47 (76) | 63 (100) | 42 (65) | 37 (56) |

SF12, SF-12 Health Survey; DTSQs, Diabetes Treatment Satisfaction Questionnaire status version, DTSQc, Diabetes Treatment Satisfaction Questionnaire change version

eTable 15. Baseline device derived physical activity dependent on wear time

| All min | 4-day (3WD, 1WE) | | 3-day (2WD, 1WE) | | 3-day (any days) | | 1-day | |  |
| --- | --- | --- | --- | --- | --- | --- | --- | --- | --- |
|  | Active control,  Mean (SD) | MOTIVATE-T2D,  mean (SD) | Active control,  Mean (SD) | MOTIVATE-T2D,  mean (SD) | Active control,  Mean (SD) | MOTIVATE-T2D,  mean (SD) | Active control,  Mean (SD) | MOTIVATE-T2D,  mean (SD) | |
| Total PA | 1484 (574) | 1393 (490) | 1484 (574) | 1393 (490) | 1505 (581) | 1372 (497) | 1498 (581) | 1351 (511) | |
| Light PA | 980 (406) | 966 (315) | 980 (406) | 966 (315) | 1001 (434) | 959 (322) | 1008 (434) | 945 (329) | |
| Moderate PA | 490 (259) | 420 (203) | 490 (259) | 420 (203) | 490 (259) | 406 (203) | 483 (259) | 399 (210) | |
| Vigorous PA | 14 (14) | 7 (7) | 14 (14) | 7 (7) | 14 (14) | 7 (7) | 7 (14) | 7 (7) | |
| MVPA | 504 (273) | 427 (203) | 504 (273) | 427 (203) | 504 (266) | 413 (210) | 490 (266) | 406 (210) | |
| MVPA10+ | 105 (168) | 49 (77) | 105 (168) | 49 (77) | 112 (175) | 49 (77) | 105 (168) | 49 (77) | |

WD, week day; WE, weekend day; PA, physical activity; MVPA, moderate-to-vigorous intensity PA; MVPA10+, MVPA accumulated in bout ≥10 minutes; All PA data is ≥16h wear time

eTable 16. Between group differences at 6- and 12-months follow-up for device derived physical activity dependent on wear time

|  | 4-day (3WD, 1WE) | | 3-day (2WD, 1WE) | | 3-day (any days) | | 1-day | |  |
| --- | --- | --- | --- | --- | --- | --- | --- | --- | --- |
|  | 6-months follow-up | 12-months follow-up | 6-months follow-up | 12-months follow-up | 6-months follow-up | 12-months follow-up | 6-months follow-up | 12-months follow-up |  |
| Total PA | -28  (-287 to 231) | -105  (-343 to 126) | -21  (-280 to 231) | -98  (-329 to 133) | 35  (-210 to 287) | -42  (-266 to 182) | -35  (-273 to 196) | -49  (-259 to 168) | |
| Light PA | -7  (-196 to 182) | -70  (-238 to 105) | -7  (-196 to 182) | -70  (-245 to 98) | 28  (-161 to 210) | -35  (-203 to 126) | -21  (-196 to 154) | -35  (-189 to 119) | |
| Moderate PA | -21  (-112 to 70) | -21  (-105 to 70) | -14  (-105 to 77) | -7  (-91 to 77) | 21  (-70 to 105) | 7  (-70 to 84) | -14  (-98 to 70) | 0  (-70 to 77) | |
| Vigorous PA | 0  (-7 to 7) | -7  (-14 to 0) | 0  (-7 to 7) | -7  (-14 to 7) | 0  (-7 to 7) | 0  (-14 to 7) | 0  (-7 to 7) | -7  (-14 to 7) | |
| MVPA | -21  (-112 to 77) | -21  (-112 to 70) | -14  (-112 to 84) | -7  (-91 to 84) | 21  (-70 to 105) | 7  (-70 to 91) | -14  (-98 to 70) | 0  (-77 to 77) | |
| MVPA10+ | 35  (-21 to 91) | 14  (-35 to 63) | 35  (-21 to 91) | 21  (-28 to 70) | 42  (-7 to 91) | 21  (-21 to 63) | 35  (-14 to 77) | 28  (-14 to 70) |  |

WD, week day; WE, weekend day; PA, physical activity; MVPA, moderate-to-vigorous intensity PA; MVPA10+, MVPA accumulated in bout ≥10 minutes;

eTable 17. Baseline continous glucose monitoring dependent on wear time

| All min | 14-day | | 10-day | | 7-day | |
| --- | --- | --- | --- | --- | --- | --- |
|  | Active control,  Mean (SD) | mHealth,  mean (SD) | Active control,  Mean (SD) | mHealth,  mean (SD) | Active control,  Mean (SD) | mHealth,  mean (SD) |
| Time in range, % (3.9-10mmol/L) | 80 (27) | 80 (27) | 80 (26) | 82 (27) | 81 (25) | 82 (27) |
| Time in tight range, % (3.9-7.8mmol/L) | 62 (27) | 64 (29) | 62 (28) | 67 (29) | 63 (28) | 67 (29) |
| Time below range, % (<3.9mmol/L) | 4 (9) | 4 (9) | 4 (9) | 4 (8) | 4 (9) | 4 (8) |
| Time below range L2, % (<3.0mmol/L) | 1 (3) | 1 (3) | 1 (3) | 1 (3) | 1 (3) | 1 (3) |
| Time above range, % (>10.0mmol/L) | 16 (27) | 15 (28) | 16 (26) | 14 (27) | 15 (26) | 14 (27) |
| Time above range L2, % (>13.9mmol/L) | 6 (17) | 7 (19) | 5 (16) | 6 (19) | 5 (16) | 6 (19) |
| Coefficient of variation, % | 25 (5) | 23 (5) | 24 (5) | 23 (5) | 24 (5) | 23 (5) |
| SD of mean glucose | 1.8 (0.7) | 1.7 (0.7) | 1.8 (0.6) | 1.7 (0.7) | 1.8 (0.6) | 1.7 (0.7) |
| Mean Glucose, mmol/L | 7.6 (2.7) | 7.5 (3.1) | 7.5 (2.7) | 7.4 (3.0) | 7.5 (2.6) | 7.4 (3.0) |

eTable 18. Between group differences at 6- and 12-months follow-up for continous glucose monitoring dependent on wear time

| All min | 14-day | | 10-day | | 7-day | |
| --- | --- | --- | --- | --- | --- | --- |
|  | 6-months follow-up | 12-months follow-up | 6-months follow-up | 12-months follow-up | 6-months follow-up | 12-months follow-up |
| Time in range, % (3.9-10mmol/L) | 3 (-7 to 12) | 6 (-5 to 16) | 3 (-6 to 12) | 6 (-4 to 16) | 3 (-6 to 11) | 4 (-5 to 13) |
| Time in tight range, % (3.9-7.8mmol/L) | 2 (-8 to 12) | 5 (-5 to 16) | 2 (-8 to 11) | 5 (-6 to 15) | 1 (-8 to 11) | 4 (-6 to 14) |
| Time below range, odds ratio ^b^ (<3.9mmol/L) | 1 (0 to 4) | 3 (1 to 14) | 1 (0 to 5) | 3 (1 to 10) | 1 (0 to 5) | 2 (1 to 9) |
| Time below range L2, odds ratio ^b^ (<3.0mmol/L) | 9  (2 to 40) | 1 (0 to 3) | 10  (2 to 43) | 1 (0 to 2) | 9  (2 to 35) | 1  (0 to 2) |
| Time above range, % (>10.0mmol/L) | -6 (-15 to 3) | -6 (-16 to 4) | -6 (-14 to 3) | -6 (-15 to 4) | -6 (-14 to 3) | -4 (-12 to 5) |
| Time above range L2, odds ratio ^b^ (>13.9mmol/L) | 1 (0 to 3) | 2 (0 to 9) | 1 (0 to 4) | 2 (0 to 7) | 1 (0 to 4) | 2 (1 to 8) |
| Coefficient of variation, % | 1 (0 to 3) | 0 (-2 to 2) | 2 (0 to 3) | 0 (-2 to 2) | 1 (0 to 3) | 0 (-1 to 2) |
| SD of mean glucose | 0 (-11 to 12) | -2 (-13 to 12) | 0 (-11 to 11) | -2 (-13 to 11) | 0 (-10 to 11) | 0 (-10 to 12) |
| Mean Glucose, % change ^a^ | -6 (-15 to 3) | -1 (-11 to 9) | -6 (-14 to 2) | -1 (-10 to 10) | -5 (-13 to 3) | -1 (-10 to 8) |

^a^ Log-transformed, interpret effect estimates as percent change. ^b^ Data were analyzed using mixed effects binomial regression, interpret effect estimates as odds ratios

eTable 19. Attendance at exercise counselling meetings

|  | Active Control,  N (%) | MOTIVATE-T2D,  N (%) |
| --- | --- | --- |
| EC1 | 64 (100) | 61 (100) |
| EC2 | 64 (100) | 59 (97) |
| EC3 | 58 (91) | 56 (92) |
| EC4 | 55 (86) | 55 (90) |
| EC5 | 53 (83) | 54 (89) |

EC, exercise counselling meeting

eTable 20. Estimated intervention delivery costs

| Staff Time ^a^ (min) | Active Control | | MOTIVATE-T2D | |
| --- | --- | --- | --- | --- |
|  | UK | Canada | UK | Canada |
| Exercise Counselling 1 | 30 | | 30 | |
| Exercise Counselling 2 | 30 | | 50 | |
| Exercise Counselling 3 | 25 | | 25 | |
| Exercise Counselling 4 | 25 | | 25 | |
| Exercise Counselling 5 | 25 | | 25 | |
| Exercise Counselling Total | 135 | | 155 | |
| Watch set up | - | | 15 | |
| Sending text messages | 5 | | 105 | |
| Exercise programming | 60 | | 45 | |
| Overall Total Counsellor Time | 320 | | 320 | |
| Estimate Total Counsellor Cost per patient | £120.00 ^b^ | $137.13 ^c^ | £192.00 ^b^ | $219.41 ^c^ |
| Watch ^d^ | - | - | £116.33 | $288.00 |
| Postage ^e^ | £3.45 | $22.73 | £4.92 | $22.73 |
| Text messages ^f^ | £0.96 | $1.92 | £2.00 | $2.00 |
| Other resource use/ costs ^g^ | | | | |
| Website ^h^ | £1.68 | $2.76 | £1.68 | $2.76 |
| Video calling subscription ^i^ | £1.19 | $2.00 | £1.19 | $2.00 |
| Video editing subscription ^j^ | £0.90 | $1.48 | £0.90 | $1.48 |
| Printing costs ^k^ | £0.75 | $1.25 | £0.74 | $1.22 |
| Envelopes/ Delivery box ^k^ | £0.06 | $0.1 | £1.56 | $2.57 |
| Estimated total delivery cost of the mHealth  intervention | £128.99 | $169.37 | £321.32 | $542.17 |

^a^ Mean delivery time has been rounded up to nearest 5 minutes. ^b^ Staff grade equivalent to NHS Agenda for Change band 5 (staff salary at £25,023 per annum), from Curtis and Burns, Unit Costs of Health and Social Care 2020, p125. Based on. Estimated cost per hour = £36 (Curtis and Burns, 2020); Includes salary, salary on costs, overheads (management costs and non-staff costs (including travel/transport)), capital overheads, and excludes costs for qualifications. ^c^ Staff grade equivalent to a dietician (2020), estimated cost per hour = $41.14 (O’Reilly et al., 2022). ^d^ Based on using a Polar Ignite 1, Polar retailer price list, without taxes (prices relevant for 2020). ^e^ UK - Royal Mail, Canada - regional and national postage averages (prices relevant to 2021). ^f^ Based on mean of 50 texts per patient at £0.04/ $0.04 per message, via online system. ^g^ These costs are distributed across the first 100 participants receiving the intervention. ^h^ Yearly Unlimited Premium Plan accessed through Wix (Wix.com), including domain name (prices relevant for 2020). ^i^ Yearly access to Zoom Pro (prices relevant to 2020). ^j^ Yearly subscription to online video editing service (prices relevant to 2020). ^k^ Price is quoted per item when 100 items ordered.

eTable 21. Wider healthcare and societal utilisation at 6- and 12-month

|  | Active control  Mean (SD) | | MOTIVATE-T2D  Mean (SD) | |
| --- | --- | --- | --- | --- |
|  | 6-month, N=44 | 12-month, N=40 | 6-month, N=49 | 12-month, N=46 |
| Secondary Care |  |  |  |  |
| A&E | 0.27 (0.73) | 0.10 (0.30) | 0.00 (0.00) | 0.09 (0.35) |
| Inpatient | 0.02 (0.15) | 0.00 (0.00) | 0.02 (0.14) | 0.02 (0.15) |
| Day Hospital | 0.07 (0.45) | 0.03 (0.16) | 0.06 (0.32) | 0.04 (0.29) |
| Clinic | 0.27 (0.54) | 0.13 (0.52) | 0.33 (0.90) | 0.26 (0.80) |
| Other | 0.05 (0.21) | 0.03 (0.16) | 0.35 (0.97) | 0.38 (1.54) |
| Total Secondary Care | 0.68 | 0.29 | 0.76 | 0.79 |
| Primary Care |  |  |  |  |
| GP (Home) | 0.00 (0.00) | 0.10 (0.44) | 0.08 (0.34) | 0.11 (0.48) |
| GP (Clinic) | 0.23 (0.68) | 0.15 (0.43) | 0.12 (0.44) | 0.35 (0.99) |
| GP (Phone) | 0.39 (0.97) | 0.35 (0.82) | 0.31 (0.82) | 0.24 (0.74) |
| Community doctor (Home) | 0.00 (0.00) | 0.13 (0.79) | 0.00 (0.00) | 0.04 (0.29) |
| Community doctor (Clinic) | 0.00 (0.00) | 0.02 (0.14) | 0.00 (0.00) | 0.04 (0.29) |
| Community doctor (phone) | 0.00 (0.00) | 0.00 (0.00) | 0.00 (0.00) | 0.30 (2.06) |
| District nurse (home) | 0.00 (0.00) | 0.00 (0.00) | 0.00 (0.00) | 0.00 (0.00) |
| District nurse (clinic) | 0.02 (0.15) | 0.00 (0.00) | 0.00 (0.00) | 0.00 (0.00) |
| District nurse (phone) | 0.00 (0.00) | 0.00 (0.00) | 0.00 (0.00) | 0.00 (0.00) |
| Practice nurse (home) | 0.00 (0.00) | 0.08 (0.35) | 0.00 (0.00) | 0.02 (0.15) |
| Practice nurse (clinic) | 0.16 (0.91) | 0.08 (0.35) | 0.04 (0.20) | 0.02 (0.15) |
| Practice nurse (phone) | 0.00 (0.00) | 0.05 (0.32) | 0.00 (0.00) | 0.00 (0.00) |
| Specialist nurse (home) | 0.00 (0.00) | 0.00 (0.00) | 0.00 (0.00) | 0.00 (0.00) |
| Specialist nurse (clinic) | 0.09 (0.36) | 0.03 (0.16) | 0.04 (0.20) | 0.00 (0.00) |
| Specialist nurse (phone) | 0.11 (0.49) | 0.08 (0.27) | 0.06 (0.24) | 0.04 (0.21) |
| Physiotherapist (home) | 0.00 (0.00) | 0.00 (0.00) | 0.00 (0.00) | 0.20 (0.93) |
| Physiotherapist (clinic) | 0.27 (1.09) | 0.00 (0.00) | 0.08 (0.40) | 0.15 (0.76) |
| Physiotherapist (phone) | 0.18 (1.21) | 0.00 (0.00) | 0.00 (0.00) | 0.04 (0.29) |
| Occupational therapist (clinic) | 0.00 (0.00) | 0.00 (0.00) | 0.00 (0.00) | 0.11 (0.74) |
| Paramedic | 0.00 (0.00) | 0.05 (0.22) | 0.00 (0.00) | 0.00 (0.00) |
| Total Primary Care | 1.45 | 1.12 | 0.73 | 1.66 |
| Social Care |  |  |  |  |
| Private home help/cleaner | 0.00 (0.00) | 0.00 (0.00) | 0.18 (1.29) | 0.13 (0.88) |
