# Supplementary material for: Mobile Health Biometrics to Enhance Exercise and Physical Activity Adherence in Type 2 Diabetes (MOTIVATE-T2D): a decentralised feasibility randomised controlled trial delivered across the UK and Canada
Source: BMJ Open. 2025 Mar 26;15(3):e092260. doi: 10.1136/bmjopen-2024-092260 (PMC12004491; doi:10.1136/bmjopen-2024-092260)
Supplement: Supplementary file 2 [file bmjopen-2024-092260.reviewer_comments.pdf]

PEER REVIEW HISTORY

BMJ Open publishes all reviews undertaken for accepted manuscripts. Reviewers are asked to complete a checklist review form and are provided with free text boxes to elaborate on their assessment. These free text comments are reproduced below.

ARTICLE DETAILS

Title (Provisional)

Mobile Health Biometrics to Enhance Exercise and Physical Activity Adherence in Type 2 Diabetes (MOTIVATE-T2D): a Decentralised Feasibility Randomised Controlled Trial Delivered Across the UK and Canada

Authors

Hesketh, Katie; Low, Jonathan; Andrews, Robert; Blitz, Sandra; Buckley, Benjamin; Falkenhain, Kaja; Job, Jennifer; Jones, Charlotte A.; Jones, Helen; Jung, Mary E.; Little, Jonathan; Mateus, Ceu; Percival, Sarah L.; Pulsford, Richard; Russon, Catherine L.; Singer , Joel; Sprung, Victoria S; McManus, Alison M; Cocks, Matthew

---

VERSION 1 - REVIEW

**Reviewer**	**1**
**Name**	**Munoz, Antonio**
**Affiliation**	**Instituto de Investigación Sanitaria Aragón**
**Date**	**10-Oct-2024**
**COI**	**None**

This is an article titled Mobile Health Biometrics to Enhance Exercise and Physical Activity Adherence in Type 2 Diabetes (MOTIVATE-T2D): a Feasibility Randomised Controlled Trial" for BMJ Open. I think this is a very interesting study, which may be of great importance for the future. I believe it is a topic of great interest to the journal's readership. It is very well written and drafted, so from my point of view, I see no need to make substantial changes to the work presented. I can therefore only congratulate the authors on their excellent work.

**Reviewer**	**2**
**Name**	**Faulkner, Melissa**
**Affiliation**	**Georgia State University, School of Nursing**
**Date**	**13-Jan-2025**
**COI**	**None**

This is a well-written manuscript that describes the feasibility of a mHealth physical activity intervention for adults recently diagnosed with type 2 diabetes using a randomized control trial. The major aims of study addressed recruitment, retention, acceptability and if the subjects in the intervention group that were using wearables would have an effect on clinical outcomes, particularly HbA1c and blood pressure. The study was well-designed and incorporated sites in both the UK and Canada.

The background and potential impact for the study were well delineated with appropriate citations to support the study design and implementation. The study methods, results, discussion. limitations and directions for future research development are comprehensive.

Some suggestions for additional details and for presenting information in the tables are provided here.

1. Clarify the inclusion of n=135 eligible subjects that expressed interest in the study noted in the abstract with the statement that n=596 expressed interest in the Consort table. This is confusing.

2. Page 10, line 5 - provide a brief description of DESMOND and X-PERT since these may not be known by others. Can the study website URL be included?

3. Page 11 - What are the numbers 28, 136, and 181 in reference to the National Institute for Healthcare Excellence Guidance?

4. How were the numbers of subjects for the interviews at select time points to gain understanding of acceptability determined? Who performed the interviews? Were the interviews recorded and transcribed? By whom? and Who did the qualitative analysis for themes and excerpts?

5. Table 1. The different components of the study groups are difficult to discern. Using columns may aid in clarity. For example, is behavioral counseling shared? It appears so since it covers the width of the table.

6. The paragraph describing the clinical outcomes is not easy to follow. Using a table to show when specific measures were collected would be helpful.

7. Pages 14 and 15: Revise the language regarding primary and secondary outcomes to state what was obtained rather than stating that they "have been proposed."

8. Include a brief statement on the psychometrics of the questionnaires that were used in the study (such as reliability and validity).

9. Describe the protocol for blood specimen collection using "home-based solutions."

10. There are numerous eTables. Are separate Consort flow charts necessary for the separate study sites?

11. Place the respective legend with figures and tables.

VERSION 1 - AUTHOR RESPONSE

Reviewer Comment	Response
**Reviewer 1**	
We thank the reviewer for taking the time to review our manuscript and for their evaluation. We value the kind words.	
**Reviewer 2**	
Thank you the reviewer for their thoughtful and encouraging feedback on our study and manuscript. We appreciate their positive assessment of our work, and we're pleased to hear that they found the design, writing, and presentation acceptable.	
Clarify the inclusion of n=135 eligible subjects that expressed interest in the study noted in the abstract with the statement that n=596 expressed interest in the Consort table. This is confusing.	We have changed the first line of the abstract 'results' section to match the Consort table with the new wording. "n=135 eligible participants expressed an interest in the trial."
Page 10, line 5 - provide a brief description of DESMOND and X-PERT since these may not be known by others. Can the study website URL be included?	Page 9 line 53: We have added a brief description of the programmes alongside URLs to the relevant information pages.<br><br>Unfortunately, the study website is no longer available as funding no longer allows access to the domain name.
Page 11 - What are the numbers 28, 136, and 181 in reference to the National Institute for Healthcare Excellence Guidance?	Page 10 line 52: National Institute for Healthcare Excellence Guidance (NICE) 28, 136 and 181 refer to specific guidelines on the management of diabetes (28), hypertension (136) and lipid modification (181). Information outlining the specific guidance has been added.
How were the numbers of subjects for the interviews at select time points to gain understanding of acceptability determined? Who performed the interviews? Were the interviews recorded and transcribed? By whom? and Who did the qualitative analysis for themes and excerpts?	Page 11 line 12: The study used a purposeful sampling framework (considering age, gender and country) which is now outlined on page 9<br><br>Page 16 line 28: Information on how interviews were recorded and transcribed, alongside information on who conducted and analysed them has been added to page 16. Briefly, interviews were conducted by BJRB, SCP and JJ who were not otherwise involved in intervention or study delivery.

	All of these researchers have expertise in qualitative research. Analysis was conducted by SCP and JJ, under the supervision of MEJ.
Table 1. The different components of the study groups are difficult to discern. Using columns may aid in clarity. For example, is behavioral counseling shared? It appears so since it covers the width of the table.	Thank you for highlighting this. We have 'unmerged' the Behavioural Counselling cells and made the content fall into the 'Shared Features' column as this was an element that was applied to both groups. We have also added borders to the columns and rows to further clarify the intervention elements in the table.
The paragraph describing the clinical outcomes is not easy to follow. Using a table to show when specific measures were collected would be helpful.	We agree with the reviewer that the clarity of the clinical outcomes section could be improved. However, we believe that a figure rather than a table showing the study timeline is best placed to do this. Unfortunately, BMJ guidelines only allow eight figures/tables so we have had to add the additional figure (eFigure 1) to the supplemental files. Page 14 line 58
Pages 14 and 15: Revise the language regarding primary and secondary outcomes to state what was obtained rather than stating that they "have been proposed."	Page 14 line 59: The term proposed has been used as the aim of this feasibility study was to evaluate potential clinical outcomes for a future RCT. However, we have changed the wording to hopefully improve clarity outlining that these measures were collected in the current trial.
Include a brief statement on the psychometrics of the questionnaires that were used in the study (such as reliability and validity).	The psychometrics of the questionnaires were included in the study protocol paper. As such, our aim is to make the current manuscript as concise as possible. Therefore, references regarding reliability and validity of the questionnaires are included.
Describe the protocol for blood specimen collection using "home-based solutions."	As above, the protocol for blood collection is outlined in detail in the corresponding protocol paper, referenced. In order to keep the current manuscript brief we have not updated this section.
There are numerous eTables. Are separate Consort flow charts necessary for the separate study sites?	We believe that maintaining separate consort flow charts for the two study sites is important for several reasons. First,

	the study sites are in different countries and we anticipate potential differences in geographical, cultural, and societal contexts that may influence recruitment, participant demographics, and other aspects of the study. By providing separate consorts, we can offer more transparent and accurate information about the enrollment, retention, and completion rates specific to each site.<br>Moreover, while we acknowledge the existence of several eTables with detailed data, the consort flow charts visually summarize key information that allows readers to quickly grasp the differences between the two sites in terms of participant flow. This separation is especially valuable for readers who may be unfamiliar with the specific challenges and contextual factors associated with each site.<br><br>We hope this clarification addresses your concern, and we are happy to revise the presentation of the consorts if needed, while maintaining this important distinction.
Place the respective legend with figures and tables.	The manuscript was formatted using BMJ open submission guidelines.
